# Exploring United States genetic counselor and healthcare interpreter perspectives: Allocation of roles within the genetic counseling encounter

**DOI:** 10.1002/jgc4.1572

**Published:** 2022-04-13

**Authors:** Melissa Wang, Marc Rosenbaum, Richard Dineen, Jessica Stoll, Karen Schmitz, Melissa Hsu

**Affiliations:** ^1^ Graduate Program in Genetic Counseling Northwestern University Feinberg School of Medicine Chicago Illinois USA; ^2^ Department of Neurology Rush University Medical Center Chicago Illinois USA; ^3^ Department of Pediatrics Rush University Medical Center Chicago Illinois USA; ^4^ Tempus Labs Chicago Illinois USA; ^5^ Rush University Cancer Center Rush University Medical Center Chicago Illinois USA; ^6^ Clinical Genetic Services Natera, Inc San Carlos California USA

**Keywords:** disparities, genetic counseling, healthcare interpreter, limited English proficiency, underrepresented populations, working relationship

## Abstract

Genetic counselors (GCs) and healthcare interpreters (HIs) are key members of the healthcare team when providing genetic counseling services to patients with Limited English Proficiency (LEP); however, the working relationship between GCs and HIs and the role each member plays within a genetic counseling session is unclear. Previous studies assessing this relationship have been qualitative and limited in sample size (Agather et al., 2018, *Journal of Genetic Counseling*, *26*, 1388; Krieger et al., 2018, *Journal of Genetic Counseling*, *26*, 1388; Lara‐Otero et al., 2019, *Health Communication*, *34*, 1608; Rosenbaum et al., 2020, *Journal of Genetic Counseling*, *29*, 352). This study utilized a quantitative approach to allow for sampling of larger populations and to simultaneously understand current perspectives of GCs and HIs regarding each other's and their own roles within a genetic counseling session. GC and HI participants from the United States were recruited via email to complete an online survey with questions regarding interactions prior to a session, roles during a session, and opportunities for collaboration and constraints in the working relationship. Descriptive and inferential statistics were utilized to analyze responses of GCs and HIs. 130 GC and 40 HI participants were included in this study. There were statistically significant differences (*p* < .001) in responses between GC and HI participants on the expected distribution of roles during a session in advocacy, psychosocial and cultural domains. Additionally, this study identified that HI desired resources and training regarding genetics and genetic counseling are currently not being met. To our knowledge, this is the largest study to simultaneously survey GC and HI perspectives on these topics. Our findings suggest the need for greater communication and collaboration between GCs and HIs to ensure high‐quality care for patients with LEP. Integrating a pre‐session meeting between the GC and HI for sessions with patients with LEP and increasing education for GCs and HIs on the roles each group brings into a session is warranted to optimize this collaborative relationship and patient care.

## Graphical abstract

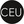

**What is known about this topic**

Genetic counselors and HIs are key members of the healthcare team when providing genetic counseling services to patients with LEP; however, the working relationship between GCs and HIs and the role each member plays within a genetic counseling session is unclear. Previous studies assessing this relationship have been qualitative and limited in sample size and generalizability.

**What this paper adds to the topic**

This is the first study to our knowledge to utilize a quantitative approach to directly compare current perspectives of GCs and HIs regarding each other's and their own roles within a genetic counseling session. This study reveals the differing opinions between GCs and HIs on who they believe roles should fall to and constraints experienced in working with one another. Key areas to improve the working relationship were also identified to provide optimal quality care for patients with LEP.

## 1 INTRODUCTION

Genetic counselors (GCs) are trained to deliver complex genetic concepts and psychosocial counseling to their patients. When working with patients with Limited English Proficiency (LEP), these components can become even more difficult to convey accurately while simultaneously assessing comprehension for English‐speaking GCs. Patients with LEP are characterized as individuals who have limited ability to read, write, speak, and understand English (Hunt & de Voogd, 2007). More than 67 million people living in the United States speak a language other than English at home, and 25 million among these individuals over the age of five years in 2019 were reported to have LEP (United States Census Bureau, 2019). Healthcare interpreters (HIs) often facilitate provider–patient communication during encounters with patients with LEP, playing an integral role in healthcare delivery.

Healthcare interpreters have traditionally been allocated to a conduit role, where their sole purpose is to interpret information in a neutral, literal manner (Dysart‐Gale, 2005). However, research suggests that healthcare provider expectations of a strictly conduit role from interpreters can silence both patients’ and interpreters’ voices, leading to compromised patient care (Hsieh & Kramer, 2012). The National Council for Interpreting in Healthcare (NCIHC) published their Standards of Practice in 2005, which outlines the multiple roles that interpreters play in healthcare: message converter, message clarifier, cultural liaison, and patient advocate (California Healthcare Interpreting Association, 2002; NCIHC, 2005). These roles are employed by the HI with careful consideration of the primary relationship between the provider and patient, and the overall health and well‐being of the patient.

Studies have found that successful medical encounters require interpreters to enact roles beyond the conduit, leading to higher levels of both patient and provider satisfaction (Dysart‐Gale, 2005; Sleptstova et al., 2014). Communication between providers and interpreters during a pre‐session meeting was recommended to establish the interpreter role in the session, define the purpose of the patient encounter, and share background information to improve the quality and understanding of the communication with the patient. A study by Gutierrez et al. (2019) also identified the critical roles HIs play as cultural mediators and facilitators of understanding for Spanish‐speaking patients during exome sequencing disclosure visits, placing healthcare interpreters in a prime position to greatly enhance the genetic counseling encounter for patients with LEP.

Conversely, the consequences of poor communication in genetic healthcare can lead to further widening of healthcare disparities and lower quality of care for patients with LEP (Ault et al., 2019; Browner et al., 2003; Cheng et al., 2018). Joseph and Guerra (2015) found that some Latina patients with LEP who received breast cancer risk counseling left the genetic counseling session with an inaccurate understanding of their risk, which could impact their subsequent adherence to risk‐reducing measures. Investigating ways to improve communication within genetic counseling sessions and retain cultural sensitivity will become increasingly important as genetic counseling becomes a more routine aspect of healthcare.

Of note, the expectations and needs of various medical providers from HIs can differ by specialty. A study by Hsieh et al. (2013) found that certain interpreter roles were particularly valued depending on the communicative needs of the providers. For instance, the authors found that nurses placed more importance on an interpreter's ability to provide emotional support to patients compared with mental health providers and oncologists. GCs were not included in this study and may have unique preferences for what roles they believe interpreters to play during a session. If the roles GCs expect of interpreters are not aligned with what interpreters believe their role to be in a patient encounter, this has the potential to lead to miscommunication of information and diminished quality of care for patients with LEP.

Descriptive experiences of GCs collaborating with interpreters within genetic counseling practice have been explored. Strategies for collaborating with HIs were identified, such as working with in‐person interpreters when possible and contracting with interpreters prior to the clinic session to ensure consistent expectations and understanding (Agather et al., 2017; Lara‐Otero et al., 2019). Similarly, the experiences of interpreters working with GCs have also been described. HIs identified a lack of resources available for interpreting in genetic counseling and challenges associated with interpreting specific genetic terminology that may not have a direct translation in the language they interpret for (Krieger et al., 2018; Lara‐Otero et al., 2019). There are also instances when culture bumps, defined as instances when one's expectations about behaviors in a certain context differ from the behaviors of individuals from a different culture, can occur within a genetic counseling session (Archer & Nickson, 2012). A recent qualitative study of HI perspectives further identified specific cultural bumps that can arise between the GC and patient with LEP in aspects of exchange of information, gender and family dynamics, and incorporation of religious and faith beliefs (Rosenbaum et al., 2020).

To decrease healthcare disparities within genetic counseling, the current expectations of both GCs and HIs regarding their roles in working with patients with LEP must be elucidated on a broader scale. Several qualitative studies have identified areas for improving patient care based on GC and HI perspectives regarding working with the other group; however, to our knowledge, none have reviewed each team member's anticipated roles in specific elements of the session, nor simultaneously compared their expectations in a quantitative manner (Agather et al., 2017; Krieger et al., 2018; Lara‐Otero et al., 2019; Rosenbaum et al., 2020). This study utilized a quantitative approach to allow for sampling of larger and broader populations, and to understand current perspectives of GCs and HIs regarding each other's and their own roles within a genetic counseling session.

The specific aims of this study were to (a) assess the collaborative relationship between HIs and GCs, (b) examine HIs’ and GCs’ perceptions of their own and each other's roles surrounding a genetic counseling session, and (c) evaluate the effectiveness of techniques used for improving communication and collaboration between HIs and GCs, as identified in previous research, to ensure optimal quality healthcare for patients with LEP.

## 2 METHODS

### 2.1 Participants and procedures

Genetic counselor participants were recruited through the National Society of Genetic Counselors (NSGC) and American Board of Genetic Counseling, Inc. (ABGC) email listservs. Eligibility for GCs was restricted to those who were board‐certified, had worked as a clinical GC within the past five calendar years (2015–2020), and had worked with a HI at least once.

Healthcare interpreter participants were initially recruited through the National Council of Interpreters in Healthcare (NCIHC) email listserv. Eligibility for HIs was restricted to individuals who had interpreted in healthcare within the past five calendar years (2015–2020). In anticipation of low response rate, both HIs who had and had not worked with GCs before were included in this sample. Due to a low initial response rate from HIs, the sampling methodology was adjusted to incorporate both snowball sampling and additional recruitment through smaller, state‐wide interpreter organizations by email (Data S1). This study was approved as exempt by the Northwestern University Institutional Review Board (IRB# STU00213378).

### 2.2 Instrumentation and procedures

Recruitment emails for GC participants were initially sent to the NSGC listserv on October 21, 2020, and the survey remained open for three weeks, until November 11, 2020. Recruitment emails for HI participants were initially sent to the NCIHC listserv on October 15, 2020, and the survey remained open just over three weeks until November 7, 2020. The HI survey deadline was extended to November 26, 2020, to recruit additional participants through state‐wide interpreter organizations who had an email address available for contact on their website (Data S1). A reminder email was sent two weeks into recruitment for both groups.

Potential participants were provided a link via the recruitment email to a survey created through the Northwestern REDCap platform. While matching in structure and content, different survey links were provided to GCs and HIs to allow for verbiage specific to the intended participants. Both surveys were developed by MW and reviewed by four board‐certified GCs. The HI survey was reviewed by an HI who met inclusion criteria. Feedback was incorporated into the survey design prior to launch of the study.

The GC and HI survey questions are included as supplemental files (Data S2; Data S3). The GC survey had 59 questions, and the HI survey had 63 questions that were both divided into four categories: interactions during pre‐session, roles during a session, opportunities and constraints in the working relationship, and demographics. The start of both surveys included mandatory screening questions based on the study inclusion criteria. The HI survey included a brief description of a GC as defined by the NSGC for the question ‘Have you worked with a genetic counselor before?’ to minimize confusion with other genetic providers (i.e., geneticist; National Society of Genetic Counselors, 2020a,b). HIs who answered ‘No’ or ‘Unsure’ to this question were provided a case scenario to aid in answering survey questions regarding roles in a session (Data S4). Survey questions that pertained to previous observations of encounters with GCs were omitted for these HI participants via skip patterns.

Survey questions regarding roles performed during sessions were divided into three domains: advocacy, psychosocial and cultural. The domains were developed by comparing the Accreditation Council for Genetic Counseling (ACGC, 2019) Core Competencies for Genetic Counselors, the NCIHC National Standards of Practice Guidelines, and previous studies exploring perceived roles of HIs (Hsieh, 2008). To quantify perspectives on the distribution of roles, GC and HI participants were asked to respond on a 6‐point Likert scale assessing who should be responsible for the role (Always GC, Mostly GC, Equally GC and HI, Neither GC nor HI, Mostly HI, and Always HI). Mann–Whitney U tests were run for each role to compare responses of GC and HI Participants. The Mann–Whitney U test is utilized to compare two independent groups when the dependent variable is measured on an ordinal scale. Survey questions exploring resources and constraints in working relationships were drawn from prior studies on these topics (Agather et al., 2017; Krieger et al., 2018; Lara‐Otero et al., 2019). Qualitative data from free‐text responses about suggestions for improving the working relationship between GCs and HIs was analyzed by the first author using qualitative content analysis to generate themes which were paired with representative quotes (Bengtsson, 2016).

Participants from each group were compensated with the opportunity to be randomly selected via raffle for one of ten available $30 VISA gift cards, for a total of twenty gift cards between both groups. Participants had the opportunity to enter the raffle at the end of the survey by providing their email address. Winners were drawn at random and contacted to receive the gift card via mail.

### 2.3 Data analysis

All statistical analyses were completed using SPSS (Version 26) for Windows. Descriptive statistics were derived to describe survey participant demographics and answer frequencies. Inferential statistics for between‐group comparisons was completed using Mann–Whitney U, chi‐squared, or Fisher's exact test. A *p*‐value of <.05 was utilized to determine statistical significance.

## 3 RESULTS

### 3.1 Participant demographics

In total, 130 GCs and 40 HIs completed the respective surveys (Table 1). Two GC participants did not complete the entire survey but were included due to responding to at least one of the main sections of questions. The response rate for GCs is challenging to calculate due to the overlapping membership of GCs between the two organizations distributing the survey. An estimated HI response rate is unable to be calculated due to the snowball sampling methodology.

**TABLE 1:** Genetic counselor and healthcare interpreter participant demographic information

	Genetic Counselor (*n* = 130) (%)	Healthcare Interpreter (*n* = 40) (%)
Age in Years (Mean)	30	49
Years Practicing (Mean)	5	12
Gender		
Female	120 (92.3)	34 (85.0)
Male	5 (3.8)	6 (15.0)
Prefer not to specify	3 (2.3)	0 (0.0)
Did not respond	2 (1.5)	0 (0.0)
Race/Ethnicity (participants could select >1)		
Asian/South Asian	8 (6.2)	10 (25.0)
Black/African American	2 (1.5)	3 (7.5)
Hispanic/Latinx	3 (2.3)	8 (20.0)
White, non‐Hispanic	115 (88.5)	17 (42.5)
Other	2 (1.5)	6 (15.0)
Prefer not to specify	2 (1.5)	0 (0.0)
Highest Level of Education		
High School Diploma	–	2 (5.0)
Some college, but no degree	–	1 (2.5)
Associate degree (i.e., AA)	–	5 (1.3)
Bachelor's Degree (i.e., BA, BS)	–	10 (25.0)
Master's Degree (i.e., MA, MS)	128 (98.5)	20 (50.0)
Professional Degree (i.e., MD)	0 (0.0)	1 (2.5)
Doctorate (i.e., PhD, EdD)	0 (0.0)	1 (2.5)
Did not respond	2 (1.5)	0 (0.0)
Region of Practice (participants could select >1)		
New England (CT, NH, ME, MA, RI, VT)	15 (11.5)	2 (5.0)
Middle Atlantic (NJ, NY, PA)	17 (13.1)	12 (30.0)
South Atlantic (DE, DC, FL, GA, MD, NC, SC, VA, WV)	24 (18.5)	1 (2.5)
North Central (IL, IN, IA, KS, MI, MN, MO, NE, ND, OH, *SD*, WI)	51 (39.2)	19 (47.5)
South Central (AL, AR, KY, LA, MS, OK, TN, TX)	17 (13.1)	1 (2.5)
Mountain (AZ, CO, ID, MT, NV, NM, UT, WY)	5 (3.8)	0 (0.0)
West (AK, CA, HI, OR, WA)	12 (9.2)	7 (17.5)
Remotely (several states)	3 (2.3)	1 (2.5)
Genetic Counseling Specialties (participants could select >1)		
Adult (non‐cancer)	29 (22.3)	9 (22.5)
Cancer	49 (37.7)	15 (37.5)
Pediatrics	65 (50.0)	18 (45.0)
Prenatal	64 (49.2)	17 (42.5)
Other	9 (6.9)	3 (7.5)
Unknown/Unsure	–	4 (10.0)
Number of times working with HI/GC		
1–10 times	16 (12.3)	17 (58.6)
11–20 times	17 (13.1)	3 (10.3)
21–30 times	12 (9.2)	2 (6.9)
31–40 times	6 (4.6)	2 (6.9)
41–50 times	12 (9.2)	2 (6.9)
51–60 times	8 (6.2)	1 (3.4)
61–70 times	5 (3.8)	0 (0.0)
71–80 times	5 (3.8)	0 (0.0)
81–90 times	3 (2.3)	0 (0.0)
91–100 times	6 (4.6)	0 (0.0)
101+ times	40 (30.8)	2 (6.9)
Languages (participants could select >1)		
American Sign Language	N/A	4 (10.0)
Amharic, Somali, or other Afro‐Asiatic languages		2 (5.0)
Arabic		5 (12.5)
Bengali		1 (2.5)
Chinese		4 (10.0)
Hindi		1 (2.5)
Nepali, Marathi, or other Indic languages		1 (2.5)
Portuguese		2 (5.0)
Punjabi		1 (2.5)
Russian		1 (2.5)
Spanish		21 (52.5)
Urdu		2 (5.0)
Vietnamese		1 (2.5)
Other		0 (0.0)
Certified/Working Toward Certification (HI)		
Yes, certified	N/A	28 (70.0)
Yes, working toward certification		6 (15.0)
No		6 (15.0)
Certification Organization (HI)		
NBCMI	N/A	7 (25.0)
CCHI		17 (60.7)
Both NBCMI and CCHI		2 (7.1)
Other		6 (21.4)

Abbreviations: CCHI, Certification Commission for Healthcare Interpreters; GC, genetic counselor; HI, healthcare interpreter; NBCMI, National Board of Certification for Medical Interpreting.

Genetic counselor participants had a mean age of 30 years and mean time practicing of five years, while HI participants had a mean age of 49 years and a mean time practicing of 12 years (Table 1). Most GC participants and HI participants, 39.2% and 47.5%, respectively, were from Region IV, categorized as the North Central states by the Census Bureau, which is approximately the Midwest (Table 1).

A total of 13 different languages were identified with Spanish being most interpreted language for HI participants at 52.5% (Table 1). No other languages were selected beyond those described in the provided list in the survey. Thirty‐four of forty (85%) HI participants were certified or working toward certification in interpreting in health care, with 17/34 (50%) through the Certification Council of Healthcare Interpreting (CCHI), 7/34 (20.6%) through the National Board for Certification in Medical Interpreting (NBCMI), and 2/34 (5.9%) having received certification through both the NBCMI and CCHI (Table 1).

### 3.2 Characterizing the working relationship

Most GC and HI participants had received or provided interpreting services for a genetic counseling session in‐person, 122/130 (93.8%) and 29/29 (100%), respectively (Data S5). Of note, 108/130 (83.1%) GC participants had also utilized interpreting services by telephone through an outside agency (Data S5). Of GC and HI participants who had received or provided interpreting services through more than one modality, 110/125 (88%) GC and 7/9 (77.7%) HI participants preferred these services to be provided in‐person (Data S5).

**FIGURE 1:**
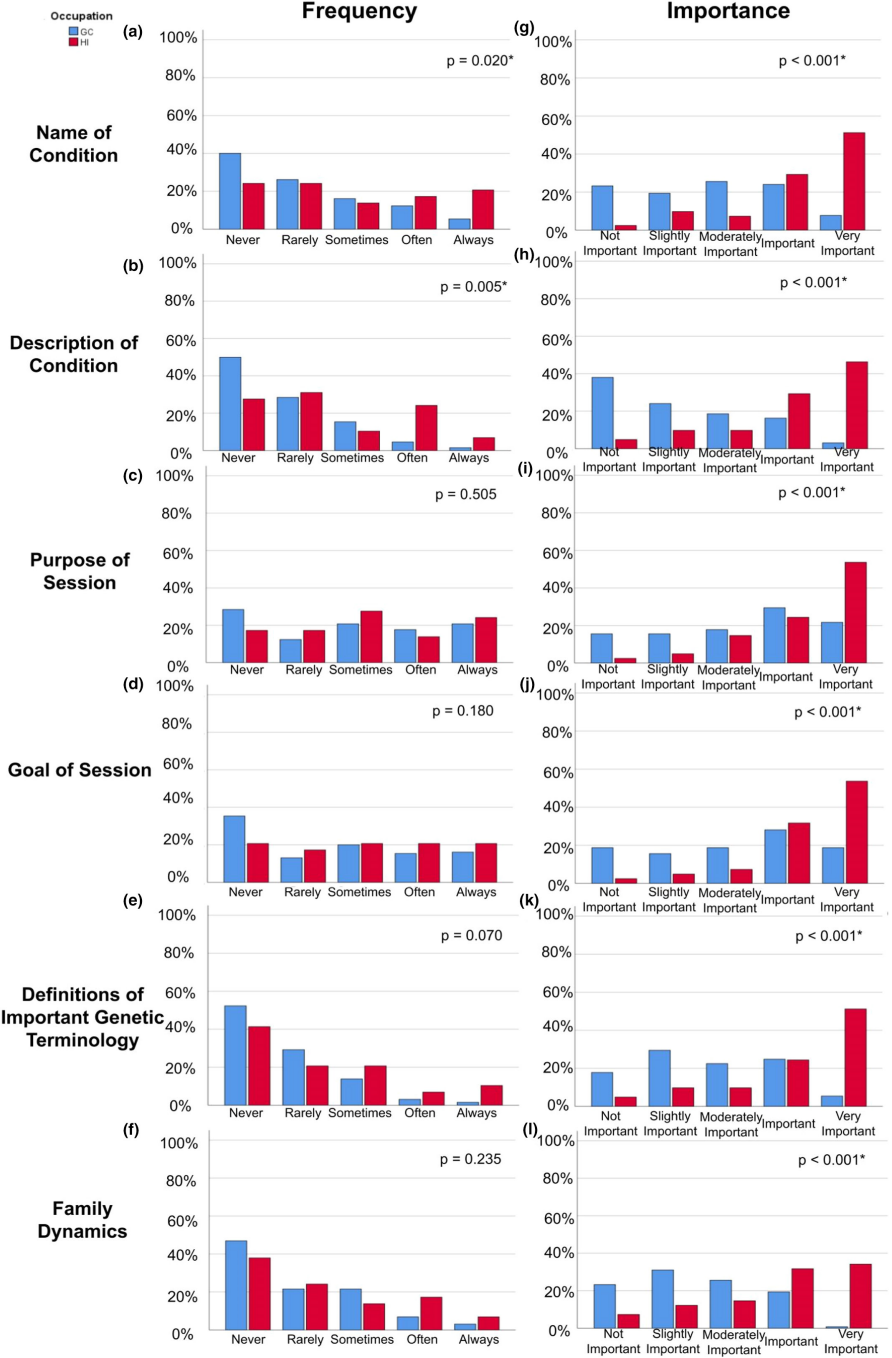
Genetic counselor and healthcare interpreter perspectives on frequency discussed and importance of topics prior to a genetic counseling session. GC, genetic counselor; HI, healthcare interpreter. Frequency x‐axis = Never, Rarely, Sometimes, Often, Always. Importance x‐axis = Not Important, Slightly Important, Moderately Important, Important, Very Important. For GCs, *n* = 128 for both questions. Y‐axis = Percent. For HIs, *n* = 29 for ‘Frequency’ and *n* = 40 for ‘Importance’. *p‐value of <.05

Regarding the frequency that various topics were discussed prior to a genetic counseling session, GC participants responded that they provided the topics ‘Name of Condition’ and ‘Description of Condition’ significantly less frequently than HI participants responded that they received this information from GCs prior to a session (Figure 1; Data S6). For all topics, there was a significant difference (*p* < .001) in responses between GC and HI participants on the importance of providing this information to an HI prior to a session, with a greater proportion of HI participants responding that providing this information prior to a session was ‘Very Important’ than GC participants (Figure 1).

### 3.3 Distribution of roles in a genetic counseling session

#### 3.3.1 Advocacy

**FIGURE 2:**
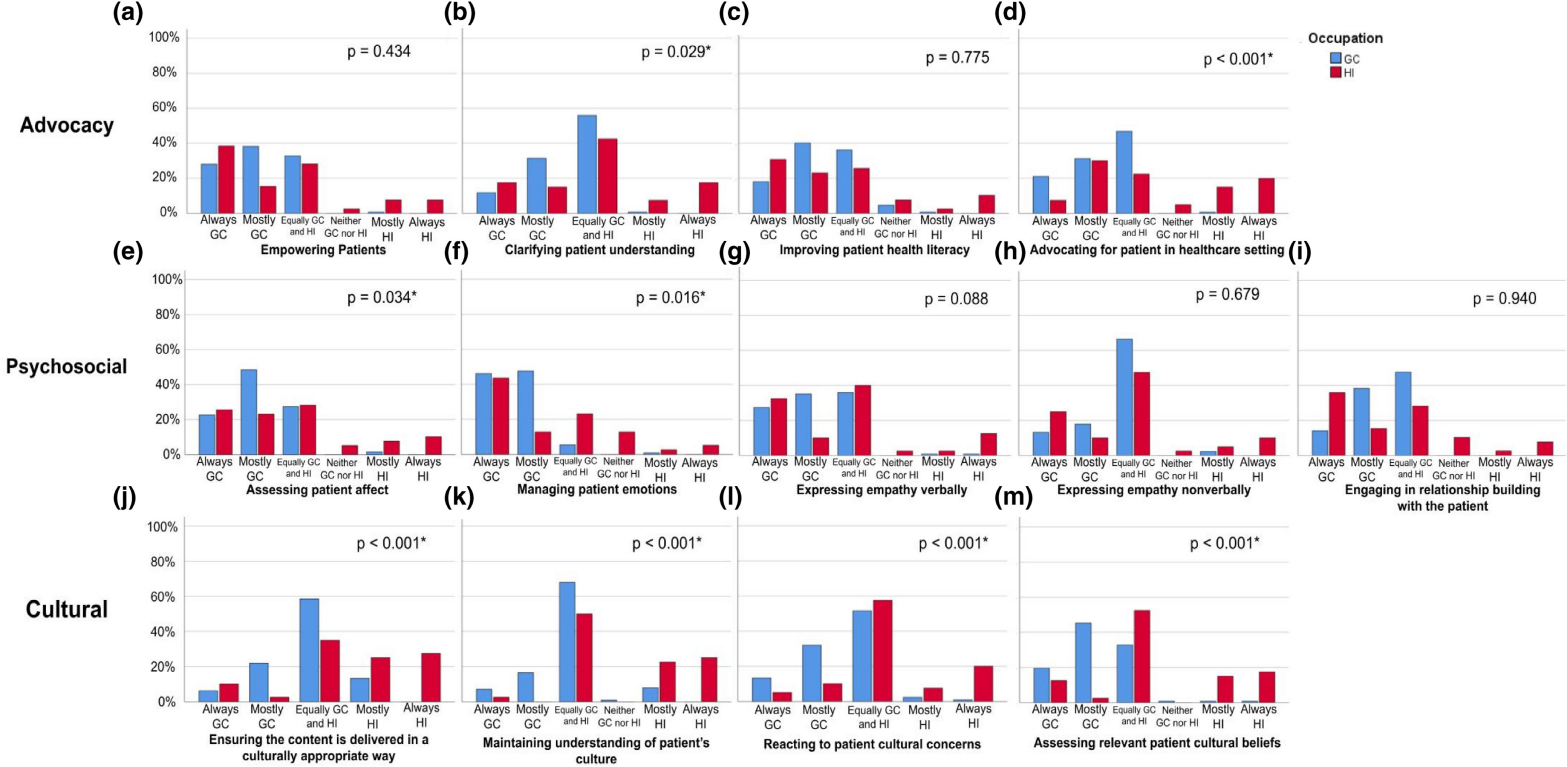
Genetic counselor and healthcare interpreter perspectives on roles during a genetic counseling session. GC, genetic counselor; HI; healthcare interpreter. X‐axis = Always GC, Mostly GC, Equally GC and HI, Neither GC nor HI, Mostly HI, Always HI. *p‐value of <.05

There was a statistically significant difference in responses between GC and HI participants on the topics of ‘Clarifying patient understanding’ (*U* = 2010.5, *p* = .029) and ‘Advocating for patient in the clinical setting’ (*U* = 1599.5, *p* < .001; Figure 2; Data S7). GC participants were more likely to respond that ‘Clarifying patient understanding’ was the responsibility of the GC, while HI participants were more likely to respond that the role was equally the responsibility of GCs and HIs. GC participants were more likely to respond that ‘Advocating for patients in the healthcare setting’ is equally the responsibility of GCs and HIs, while HIs were more likely to respond that the role was the responsibility of HIs. Of note, 6/127 (4.7%) of GC participants and 3/39 (7.7%) of HI participants responded that ‘Improving patient health literacy’ was the responsibility of neither the GC nor the HI.

#### 3.3.2 Psychosocial

There was also a statistically significant difference between GC and HI responses on the topics of ‘Assessing patient affect’ (*U* = 1968.5, *p* = .034) and ‘Managing Patient Emotions’ (*U* = 1913.0, *p* = .016; Figure 2; Data S7). GC participants were more likely to respond that the roles fell to GCs, while HIs were more likely to respond that the roles fell equally to GCs and HIs. Of note, 5/39 (12.8%) of HI participants responded that ‘Managing Patient Emotions’ was the responsibility of neither the GC nor the HI.

#### 3.3.3 Cultural

There was also a significant difference between GC and HI responses on all surveyed cultural roles: ‘Ensuring the content is delivered in a culturally appropriate way’ (*U* = 1450.0, *p* < .001), ‘Maintaining understanding of patient's culture’ (*U* = 1258.5, *p* < .001), ‘Reacting to patient cultural concerns’ (*U* = 1463.5, *p* < .001), and ‘Assessing relevant patient cultural beliefs’ (*U* = 1168.0, *p* < .001; Figure 2; Data S7). For the roles of ‘Ensuring the content is delivered in a culturally‐appropriate way’, ‘Maintaining understanding of patient's culture’, and ‘Reacting to patient cultural concerns’, GC participants were more likely to respond that cultural roles were equally the responsibility of GCs and HIs, while HI participants were more likely to respond that HIs were responsible for these roles. For the role ‘Assessing relevant patient cultural beliefs’, GCs were more likely to respond this role fell to GCs, while HIs were more likely to respond this role fell equally to GCs and HIs.

### 3.4 Opportunities and constraints in the working relationship

#### 3.4.1 Resources

**FIGURE 3:**
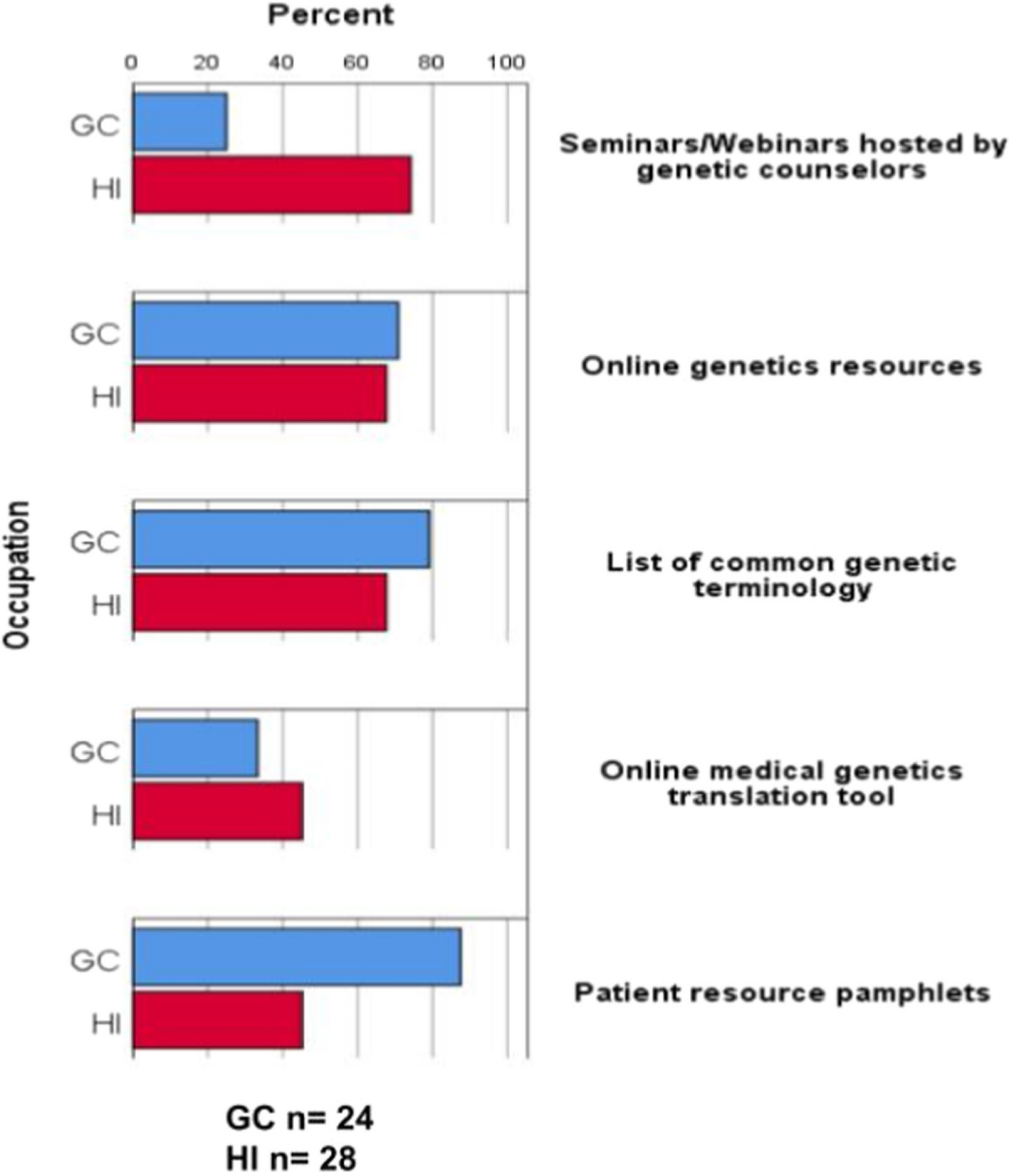
Resources provided previously by genetic counselors and desired by healthcare interpreters who had not received resources before. GC, genetic counselor; HI, healthcare interpreter

Twenty‐four of one hundred and twenty‐eight (24/128, 18.8%) GC respondents indicated that they have provided resources to HIs (Figure 3). Of those who reported they do provide these resources to HI colleagues, the most provided resource was patient resource pamphlets (21/24, 87.5%; Figure 3). Other resources provided as shared in free‐text responses included YouTube videos of genetics concepts, outline with major counseling points for specific sessions, and copies of results letters. Nine of twenty‐nine (31.0%) HI participants who have worked with a GC responded that they have received resources from a GC, with the most received resource also being patient resource pamphlets (6/9, 66.7%; Figure 3). Of HI respondents who have not previously received resources from a GC, 28/31 (90.3%) participants responded they would want to receive resources from a GC. The most desired resource was seminars/webinars hosted by a GC (23/28, 82.1%). Other desired resources as shared in free‐text responses included the goals and responsibilities GCs are taught in their training, and diagrams and photographs related to genetics.

#### 3.4.2 Constraints

**FIGURE 4:**
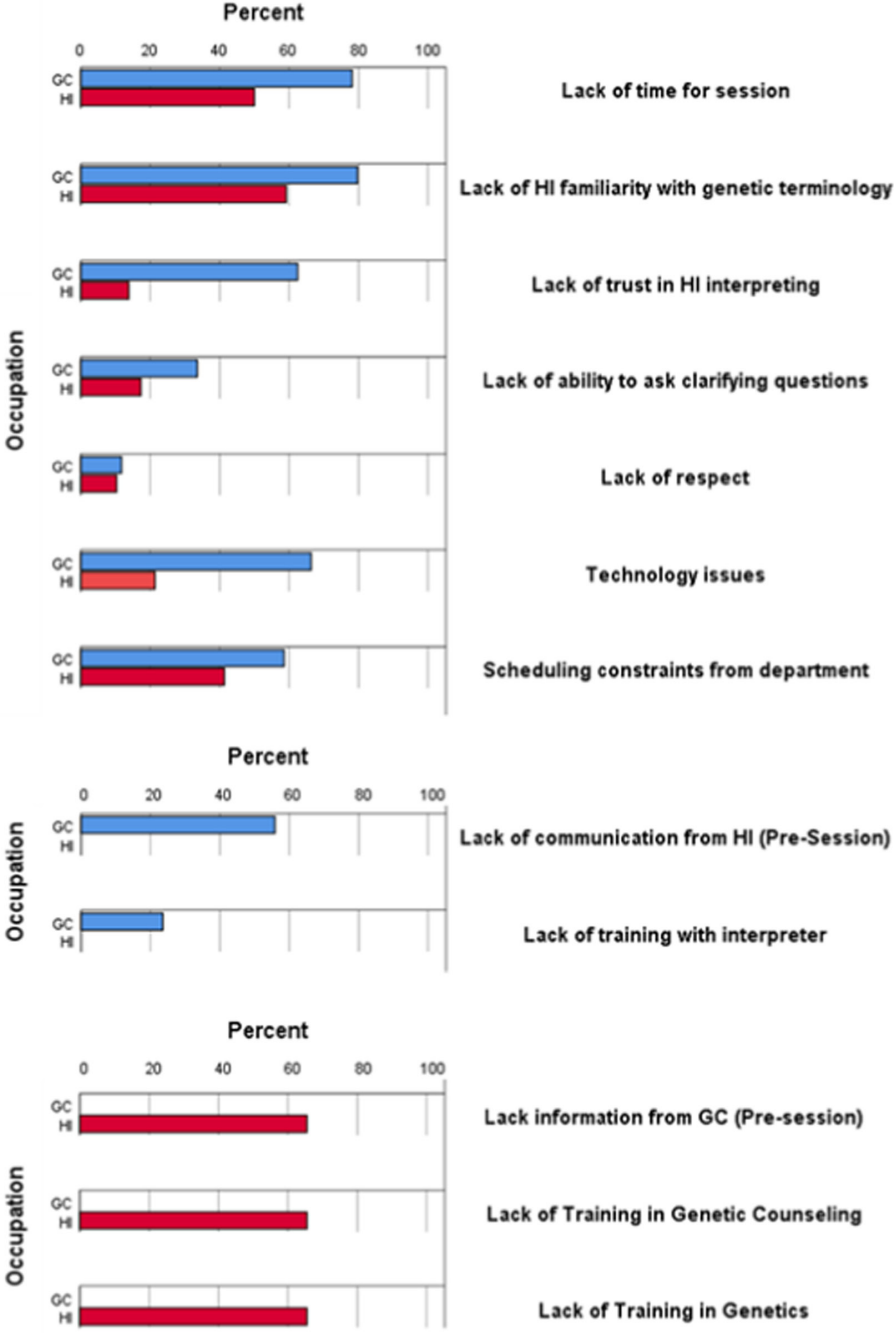
Constraints in working relationship as perceived by genetic counselors and healthcare interpreters. GC, genetic counselor. HI, healthcare interpreter

The top three constraints impacting this working relationship as indicated by GC participants were ‘lack of time for patient session’ (100/128, 78.1%), ‘HI lack of familiarity with genetic terminology’ (102/128, 79.7%), and ‘technology issues (if working with remote interpreter)’ (85/123, 69.1%; Figure 4). The three most perceived constraints by HI participants included ‘insufficient information pre‐session from genetic counselor to provide interpreting’ (19/29, 67.9%) and ‘lack of training in interpreting in genetics’ (19/29, 67.9%); these same 19 participants also indicated the constraint of ‘lack of training in interpreting in genetic counseling’ (19/29, 67.9%). Participants that marked constraints as ‘Not applicable’ were omitted from analysis.

## 4 DISCUSSION

Genetic counselors and HIs are both integral members of the care team when delivering genetic counseling services to patients with LEP. Prior studies have explored the perspectives of GCs and HIs independently and in a qualitative manner, with this study being the first to our knowledge to simultaneously compare GC and HI perspectives in a quantitative manner. The results of this study reveal that there are differences in perceptions of roles during a session and opportunities for improvement of the working relationship between GCs and HIs.

The working relationship and modalities of interactions between the samples was evaluated. Regarding the types of information discussed during a pre‐session meeting, HI participants viewed all categories of information of greater importance to discuss compared with GC participants (Figure 1). Several free‐text responses from both GC and HI participants also stated that a pre‐session would be beneficial to improving the working relationship between GCs and HIs (Data S8). This aligns with previous research which indicated that setting up a meeting between the GC and HI prior to sessions for patients with LEP was recommended by both GC and HI participants (Agather et al., 2017; Krieger et al., 2018). In‐person interpreting services were identified to be the preferred modality for receiving interpreting services by the surveyed GCs, which is also consistent with previous research (Agather et al., 2017). While only nine HI participants who had provided interpreting services for a genetic counseling session had done so through more than one modality, most of these participants stated in‐person was their preferred modality of providing interpreting services as well.

In reviewing the practice‐based competencies defined by the ACGC, the National Standards of Practice by the NCIHC and prior research on roles perceived by HIs, there is considerable overlap in roles and responsibilities between GCs and HIs within a session (Hsieh, 2008). However, results from this study suggest that GCs and HIs are not aligned in their perception of responsibilities of certain roles within a session. In general, across different roles, GCs were more likely to respond that roles were ‘Always GC’ or ‘Mostly GC’, while HIs were more varied in their responses. Several roles were identified to have a statistically significant difference in responses between GCs and HIs. Within the described advocacy roles, HIs view part of their roles as a patient advocate and to intervene if the patient's ‘dignity or safety is at risk’, while GCs may still be reticent to seeing HIs beyond the conduit role (NCIHC, 2005). This may also be different from how GCs define advocacy within a healthcare setting, leading to the observed differences in responses. Additionally, the role of ‘clarifying patient understanding’ may not function within the same context for both GCs and HIs. From the GC perspective, the impact of additional cultural factors may necessitate ‘clarifying patient understanding’ when culture bumps are more likely to occur between a GC and a patient with LEP (Rosenbaum et al., 2020). HIs, however, may be likely to consider ‘clarifying patient understanding’ a normative task, given its standard role in interlingual interaction. Thus, HIs may not regard ‘clarifying patient understanding’ as a function of advocacy within the same context as GCs.

For psychosocial roles, a higher proportion of HIs responded that ‘managing patient emotions’ falls to ‘Neither GCs nor His’ compared with other topics. Possible explanations include that HIs are not aware of the psychosocial counseling aspects that GCs are trained to value highly within a session, consistent with prior literature, or may feel psychosocial roles fall to a different provider such as a social worker. In addition, HIs may view conveying empathy under the function of accuracy, in which they aim to convey the empathic spirit of a message as close to the original as possible rather than providing empathy themselves. These results suggest that there is a need for increased education for both groups regarding the roles that GCs and HIs are trained to undertake during a session. Additional studies are required to observe how GCs and HIs collaborate within a session in practice and the outcomes of increased education for both groups regarding each other's training and roles on this working relationship and resultant impact on patient care.

Regarding cultural aspects of a session, cultural competency is highly emphasized during training for GCs, while HIs are trained to view themselves as a cultural liaison or broker. GCs and HIs responded differently for all cultural roles they were surveyed about with regards to who they believe the role should fall to. If there are cultural disconnects between the GC and the patient that are not identified and resolved by either the GC or HI, this may lead to diminished quality of care for the patient. Rosenbaum et al. (2020) found that HIs identified cultural misunderstanding between the GC and patient during genetic counseling sessions, which may have led to broken rapport and mistrust from the patient; however, who is responsible and best equipped to address these cultural misunderstandings is still unknown. Further research is required to investigate the most effective methods of managing and resolving instances of cultural discordance within a genetic counseling session.

For some roles in the Advocacy and Psychosocial domains, there appears to be some alignment between GC and HI responses in distribution of the roles; however, there remains opportunity to collaborate further to determine how to best serve patients with LEP. For instance, most GC and HI participants responded that the role of ‘Expressing empathy verbally (empathy statements and word choice)’ fell mostly to GCs. GCs may expect to initiate a statement to convey empathy to be interpreted by an HI to the patient. However, Gutierrez et al. (2019) found that Spanish HIs also employed various empathic linguistic tools during sessions to deliver genomic testing results, such as contextualization of information and encouragement. Collaborating on ways to phrase empathy statements in a way that can be easily interpreted and will resonate best with patients with LEP across various languages and cultures can be further explored. Areas where GCs and HIs responded more similarly in expectations and roles, such as empowering patients and engaging in relationship building with the patient both falling to GCs, could be starting points for improving the working relationship, subsequently improving care for patients with LEP. Additionally, different specialties in genetic counseling may emphasize or require different roles from HIs, and HIs collaborating with GCs may require different resources depending on genetic specialty. Future studies investigating the unique needs of GCs and HIs in different specialties are warranted.

This study also explored opportunities and constraints within the working relationship between GCs and HIs, which help to provide additional context to some of the previously discussed data regarding the working relationship. The results revealed that the lack of information from a pre‐session meeting was one of the most common constraints identified by HIs when working with GCs, with the majority of HIs reporting this as a constraint (Figure 4). The previously summarized data regarding the importance of discussing certain topics during a pre‐session meeting may also help to elucidate what types of information would be most helpful for interpreters to have before entering a session. ‘Technology issues (if working with a remote interpreter)’ was one of the most frequently reported constraints by GCs in this study, which may be a factor in the preference toward in‐person interpreters. Further studies on the differences between in‐person and remote interpreting services that lead to preferences toward and patient benefits of in‐person services are needed.

In addition to the lack of a pre‐session, several constraints were reported by both GCs and HIs on working with each other. Both GCs and HIs identified the lack of HI training in genetics to be one of the most common constraints (Figure 4). Lack of HI familiarity of genetic terminology can hinder the delivery of information and may cause an interactive component with other constraints surveyed, including lack of time for a patient session and lack of trust in the HI interpreting from the GC. The amount of time needed for a patient session can become extended if the HI requires additional time to clarify complex concepts prior to message delivery. Increasing the number of genetics workshops available to HIs could help to increase HI confidence within a genetic counseling encounter.

There were also several free‐text responses that described very specific negative experiences from each GCs and HIs. Having had prior negative experiences with an HI or GC may negatively impact subsequent interactions with and opinions toward that group. One of the more frequently experienced constraints by GC participants was lack of trust in interpretations made by the HI, while one of the least frequently experienced constraints by HI participants was a lack of trust from a GC of their interpreting (Figure 4). The lack of trust in HIs experienced by GCs may not often be directly conveyed to HIs; therefore, HIs are unaware that there is a lack of trust in their interpreting. Prior studies have also identified that providers often measure the amount of trust in an HI based on their linguistic ability (Hsieh et al., 2010). Some GC participants conveyed that working with the same group of HIs multiple times can help to build trust and improve the working relationship. Unfortunately, it was also stated by GCs that they frequently do not work with the same HI on more than one occasion; thus, there is a lack of ability to build a working relationship. Identifying additional factors that positively or negatively influence the working relationship between GCs and HIs may help to target efforts to resolve or minimize these constraints.

Finally, development and provision of resources may help to combat the above constraints. Only a small minority of GCs in this study have provided resources to a HI, while a majority of HIs who have not received resources from a GC responded they would want to receive these resources (Figure 3). Further, the resources most often provided by this subset of GCs were those least desired by HIs, and the resources least provided by GCs were those most desired by HIs, revealing a need for appropriate resources for HIs that is currently not being met by GCs. The resource most desired by HIs in this study was seminars/webinars hosted by GCs (Figure 3). Potential seminar topics could include what to expect during a genetic counseling encounter, definitions of common terminology used during sessions, and roles GCs are trained to undertake during a session. Further research identifying ideal topics to cover during these seminars and ways to improve HI training in genetics and genetic counseling is needed. Increased communication between GCs and HIs prior to or after a session and education of GCs regarding what resources would be helpful to HIs could improve the working relationship in effort to improve the quality of care for patients with LEP.

### 4.1 Study limitations

Our study is the largest study, to our knowledge, to directly compare GC and HI perspectives of this working relationship using a quantitative methodology; however, there were several limitations. The sample of HIs was smaller than that of GCs and included HIs who had not worked with GCs before to aid with HI sample size. In addition, all HIs participants who had interpreted for a GC had provided interpreting services for an in‐person genetic counseling session, and 65.5% had not interpreted for a GC by any other method. HIs who provide in‐person services within a hospital system are often required to obtain certification and undergo additional training within their departments. Therefore, our sample of HIs who have interpreted for a GC may be overrepresented by HIs who are certified and may have completed training programs to take the NBCMI and CCHI exams, which are standardized exams often required for HIs to work at a healthcare institution. Furthermore, a large portion of HIs was recruited from the Midwest region, which is known to have fewer organizations and available guidelines compared with other regions of the United States. Finally, while female interpreters outnumber male interpreters 7 to 3 in the interpreter workforce, male interpreters were underrepresented in our sample and there may be differences in views not captured in this study (Executive Summary, 2016). For these reasons, this data has limited generalizability to all HIs.

Regarding modalities of interpreting services, the COVID‐19 global pandemic may have increased the necessity for utilizing telephone and telehealth services that was not accounted for in this study. This potential increase in use or provision of telephone and telehealth interpreting services in a short period of time may have been unfamiliar to some participants, resulting in a preference for in‐person interpreting services.

As with most survey‐based studies, there is potential for selection bias. Participants who are more invested in the working relationship between GCs and HIs may have been more likely to participate in this study. In addition, many GC and HI participants provided responses in the free‐text sections, potentially indicating that participants recruited had unique experiences from working with HIs or GCs that they wished to share.

### 4.2 Practice implications

There are opportunities for increasing education for GCs and HIs about the roles and expectations the other party brings into a genetic counseling session. Genetic counseling programs can include more robust training for genetic counseling students regarding working with and the roles of interpreters. Increased collaboration between genetics and interpreting services departments both within a hospital system and on an individual level between a GC and HI could improve the working relationship. This could include GC provision of requested resources for interpreters, such as genetics‐focused seminars or webinars on topics such as what to expect during a genetic counseling encounter, definitions of common terminology used during sessions, and roles GCs are trained to undertake during a session. Integrating a pre‐session meeting between the GC and HI into the clinic workflow to review important details regarding the session may be beneficial to both the working relationship and quality of patient care.

## 5 CONCLUSION

GCs and HIs both play important roles within the genetic counseling session with regard to the provision of quality care to the patients they serve. The topic of the working relationship between GCs and HIs is understudied and more research is necessary to define and improve collaboration between both groups. Gaining a better understanding of the expectations toward the distribution of roles within a session is critical to defining this working relationship and improving care for patients with LEP.

The comparison between GC and HI perspectives through this quantitative study expose the differing opinions held by both groups. This is the largest study, to our knowledge, to directly compare the perspectives of both GCs and HIs, which revealed that both groups are often not aligned in their expectations regarding interactions prior to a session and roles within a session. Several key areas such as time allotted for a session, lack of trust in an HI’s interpreting, and technology issues have constrained the GC and HI working relationship and will be important areas to brainstorm and research solutions for improvement. Provision and development of resources from GCs was found to be much desired by HIs, most particularly in the form of seminars or webinars hosted by GCs. GCs may also benefit from increased training and resources in working with HIs to provide optimal care and genetic counseling to patients with LEP.

Continuing to explore and define how GCs and HIs are best able to collaborate and interact before, during, and after a genetic counseling session are important future directions for research. By increasing avenues for communication, connection, and collaboration between GCs and HIs, improvements and enhancements to patient care for patients from diverse backgrounds are bound to follow.

## AUTHOR CONTRIBUTIONS

Melissa Wang confirms that they had full access to all the data in the study and take responsibility for the integrity of the data and the accuracy of the data analysis. All of the authors gave final approval of this version to be published and agree to be accountable for all aspects of the work in ensuring that questions related to the accuracy or integrity of any part of the work are appropriately investigated and resolved.

## COMPLIANCE WITH ETHICAL STANDARDS

### Conflict of interest

Melissa Wang, Marc Rosenbaum, Richard Dineen, Karen Schmitz, and Melissa Hsu declare that they have no conflicts of interest to disclose. Jessica Stoll is an employee of Tempus Labs, Inc.

### Human studies and informed consent

This study was approved and declared exempt by the Northwestern University Institutional Review Board (IRB) in accordance with the US Federal Policy for the Protection of Human Subjects. An online consent form was utilized at the start of the survey and implied informed consent was obtained for individuals who voluntarily completed the online survey and submitted their responses.

### Animal studies

No non‐human animal studies were carried out by the authors for this article.

### Data sharing and data accessibility

The data that supports the findings of this study are available in the supplementary material of this article.

## Supporting information

Data S1

Data S2

Data S3

Data S4

Data S5

Data S6‐S7

Data S8
