# Supplementary material for: Exploring United States genetic counselor and healthcare interpreter perspectives: Allocation of roles within the genetic counseling encounter
Source: J Genet Couns. 2022 Apr 13;31(4):976–88. doi: 10.1002/jgc4.1572 (PMC9542924; doi:10.1002/jgc4.1572)
Supplement: Supplementary file 1 — Data S1 [file JGC4-31-976-s001.docx]

**Supplemental Data 1.** List of Interpreter Organizations Contacted for Distribution of Healthcare Interpreter Survey

- Austin Area Translators & Interpreters Association
- California Healthcare Interpreting Association
- Medical Interpreter Network of Georgia
- Nebraska Association for Translators and Interpreters
- Oregon Health Care Interpreters Association
- Upper Midwest Translators and Interpreters Association

Interpreter organizations listed on the National Council for Interpreting in Healthcare website (<https://www.ncihc.org/interpreter-associations>) were contacted if a valid email address was provided on their website.
