# Supplementary material for: Exploring United States genetic counselor and healthcare interpreter perspectives: Allocation of roles within the genetic counseling encounter
Source: J Genet Couns. 2022 Apr 13;31(4):976–88. doi: 10.1002/jgc4.1572 (PMC9542924; doi:10.1002/jgc4.1572)
Supplement: Supplementary file 2 — Data S2 [file JGC4-31-976-s004.pdf]

Genetic Counselor and Interpreter Perspectives: Allocation of Roles PID 5801

Codebook

Data Dictionary Codebook

12/17/2020 6:12pm

Collapse all instruments

#	Variable / Field Name	Field Label<br>*Field Note*	Field Attributes (Field Type, Validation, Choices, Calculations, etc.)
Instrument: **Gc Survey** (gc_survey) Enabled as survey			Collapse
1	record_id	Record ID	text
2	consent_gc	By selecting "I Agree", you understand the information above and agree to the terms of this study.	radio, Required<br>1 I Agree<br>Custom alignment: LV
3	screen_age	Section Header:<br>Are you 18 years of age or older?	yesno, Required<br>1 Yes<br>0 No<br>Custom alignment: LV<br>Stop actions on 0
4	screen_certified	Section Header:<br>Are you a certified genetic counselor by the American Board of Genetic Counselors or the American Board of Medical Genetics?	yesno, Required<br>1 Yes<br>0 No<br>Custom alignment: LV<br>Stop actions on 0
5	screen_workint	Section Header:<br>Have you ever worked with a health care interpreter in a genetic counseling encounter?	yesno, Required<br>1 Yes<br>0 No<br>Custom alignment: LV<br>Stop actions on 0
6	screen_yearspractice	Section Header:<br>How many years have you been a practicing clinical genetic counselor in the United States (round to the nearest whole year)?	text (integer, Min: 1, Max: 100)
7	screen_timesint	Thinking about from the start of your career practicing as a genetic counselor, approximately how many times have you worked with a healthcare interpreter?	dropdown<br>1 1-10 times<br>2 11-20 times<br>3 21-30 times<br>4 31-40 times<br>5 41-50 times<br>6 51-60 times<br>7 61-70 times<br>8 71-80 times<br>9 81-90 times<br>10 91-100 times<br>11 101+ times

<table>
<tr><td>8</td><td>specialtieswithint</td><td>Section Header:
What specialties have you worked in as a clinical genetic counselor where you have worked with an interpreter (select all that apply)?</td><td>checkbox
<table>
<tr><td>1</td><td>specialtieswithint___1</td><td>Pediatrics - childhood onset conditions, newborns in the NICU/PICU</td></tr>
<tr><td>2</td><td>specialtieswithint___2</td><td>Prenatal - pregnant women, discussion of pregnancy screening and testing options</td></tr>
<tr><td>3</td><td>specialtieswithint___3</td><td>Cancer - pediatric or adult</td></tr>
<tr><td>4</td><td>specialtieswithint___4</td><td>Adult (non-cancer) - cardiology, neurology, etc.</td></tr>
<tr><td>5</td><td>specialtieswithint___5</td><td>Other (please specify): {specialtieswithint2}</td></tr>
</table></td></tr>
<tr><td>9</td><td>specialtieswithint2</td><td></td><td>text</td></tr>
<tr><td>10</td><td>regions_gc</td><td>In which regions of the United States have you provided clinical genetic counseling (select all that apply)?</td><td>checkbox
<table>
<tr><td>1</td><td>regions_gc___1</td><td>Region 1 - New England (Connecticut, Maine, Massachusetts, New Hampshire, Rhode Island, Vermont)</td></tr>
<tr><td>2</td><td>regions_gc___2</td><td>Region 2 - Middle Atlantic (New Jersey, New York, Pennsylvania)</td></tr>
<tr><td>3</td><td>regions_gc___3</td><td>Region 3 - South Atlantic (Delaware, District of Columbia, Florida, Georgia, Maryland, North Carolina, South Carolina, Virginia, West Virginia)</td></tr>
<tr><td>4</td><td>regions_gc___4</td><td>Region 4 - North Central (Kansas, Illinois, Indiana, Iowa, Michigan, Minnesota, Missouri, Nebraska, North Dakota, Ohio, South Dakota Wisconsin)</td></tr>
<tr><td>5</td><td>regions_gc___5</td><td>Region 5 - South Central (Alabama, Arkansas, Kentucky, Louisiana, Mississippi, Oklahoma, Tennessee, Texas)</td></tr>
<tr><td>6</td><td>regions_gc___6</td><td>Region 6 - Mountain (Arizona, Colorado, Idaho, Montana, Nevada, New Mexico, Utah, Wyoming)</td></tr>
<tr><td>7</td><td>regions_gc___7</td><td>Region 7 - West (Alaska, California, Hawaii, Oregon, Washington)</td></tr>
<tr><td>8</td><td>regions_gc___8</td><td>I provide services across several states remotely.</td></tr>
</table></td></tr>
<tr><td>11</td><td>language_gc</td><td>Section Header:
For what language(s), in addition to English, do you have at least intermediate language proficiency (would be able to understand and/or counsel at least 50% of the genetic counseling session in that language)? Please select all that apply.</td><td>checkbox
<table>
<tr><td>1</td><td>language_gc___1</td><td>I do not have at least intermediate language proficiency in any other language</td></tr>
<tr><td>2</td><td>language_gc___2</td><td>American Sign Language</td></tr>
<tr><td>35</td><td>language_gc___35</td><td>Amharic, Somali, or other Afro-Asiatic languages</td></tr>
<tr><td>3</td><td>language_gc___3</td><td>Arabic</td></tr>
<tr><td>4</td><td>language_gc___4</td><td>Armenian</td></tr>
<tr><td>5</td><td>language_gc___5</td><td>Bengali</td></tr>
<tr><td>6</td><td>language_gc___6</td><td>Chinese (incl. Mandarin, Cantonese)</td></tr>
</table></td></tr>
</table>

7	language_gc___7	French (incl. Cajun)
8	language_gc___8	German
9	language_gc___9	Greek
10	language_gc___10	Gujarati
11	language_gc___11	Haitian
12	language_gc___12	Hebrew
13	language_gc___13	Hindi
14	language_gc___14	Hmong
36	language_gc___36	Ilocano, Samoan, Hawaiian, or other Austronesian languages
15	language_gc___15	Italian
16	language_gc___16	Japanese
17	language_gc___17	Khmer
18	language_gc___18	Korean
37	language_gc___37	Malayalam, Kannada, or other Dravidian languages
19	language_gc___19	Navajo
38	language_gc___38	Nepali, Marathi, or other Indic languages
20	language_gc___20	Persian (incl. Farsi, Dari)
21	language_gc___21	Polish
22	language_gc___22	Portuguese
23	language_gc___23	Punjabi
24	language_gc___24	Russian
25	language_gc___25	Serbo-Croatian
26	language_gc___26	Spanish
27	language_gc___27	Swahili or other languages of Central, Eastern, and Southern Africa
28	language_gc___28	Tagalog (incl. Filipino)
29	language_gc___29	Tamil
30	language_gc___30	Telugu
39	language_gc___39	Thai, Lao, or other Tai-Kadai languages
31	language_gc___31	Ukrainian or other Slavic languages
32	language_gc___32	Urdu
33	language_gc___33	Vietnamese
40	language_gc___40	Yiddish, Pennsylvania Dutch or other West Germanic languages
41	language_gc___41	Yoruba, Twi, Igbo, or other languages of Western Africa
34	language_gc___34	Other (please specify): {languagebox_gc}

Custom alignment: LV
Field Annotation: @NONEOFTHEABOVE=1

12	languagebox_gc		text

13	methodsint_gc	Section Header:<br>Which of the following methods have you employed to utilize interpreting services in the past? Please select all that apply:	checkbox<br>1 methodsint_gc___1 In-person interpreter<br>2 methodsint_gc___2 Telephone interpreter through hospital (in-house)<br>3 methodsint_gc___3 Telehealth (with audio and video) interpreter through hospital (in-house)<br>4 methodsint_gc___4 Telephone interpreter through outside agency<br>5 methodsint_gc___5 Telehealth (with audio and video) interpreter through outside agency<br>6 methodsint_gc___6 Other (please specify): {methodsintbox_gc}
14	methodsintbox_gc		text
15	methodsintpref_gc	Of the methods you have utilized interpreting services in the past, what is your preferred method of receiving interpreting services?	radio<br>1 In-person interpreter<br>2 Telephone interpreter through hospital (in-house)<br>3 Telehealth (with audio and video) interpreter through hospital (in-house)<br>4 Telephone interpreter through outside agency<br>5 Telehealth (with audio and video) interpreter through outside agency<br>6 Other (please specify): {methodsintprefbox_gc}
16	methodsintprefbox_gc		text
17	name	Section Header: *How often do you provide to an interpreter before a session the following information:*<br>The name of the condition the patient has or is at risk for	radio (Matrix)<br>1 Never<br>2 Rarely<br>3 Sometimes<br>4 Often<br>5 Always
18	descript	A description of the condition (i.e. main physical features, gene(s) involved, etc.)	radio (Matrix)<br>1 Never<br>2 Rarely<br>3 Sometimes<br>4 Often<br>5 Always
19	purpose	The purpose of the session (i.e. reason for referral)	radio (Matrix)<br>1 Never<br>2 Rarely<br>3 Sometimes<br>4 Often<br>5 Always
20	goal	The goal of the session (i.e. to discuss genetic testing options, to give genetic testing results, etc.)	radio (Matrix)<br>1 Never<br>2 Rarely<br>3 Sometimes<br>4 Often<br>5 Always

21	definition	Definitions of important terminology	radio (Matrix)<br>1 Never<br>2 Rarely<br>3 Sometimes<br>4 Often<br>5 Always
22	famdynam	Family dynamics (i.e. child is adopted, parents are divorced, legal guardian is the grandmother, etc.)	radio (Matrix)<br>1 Never<br>2 Rarely<br>3 Sometimes<br>4 Often<br>5 Always
23	name_imp	Section Header: *How important do you think it is to provide to an interpreter before a session the following information:*<br>The name of the condition the patient has or is at risk for	radio (Matrix)<br>1 Not Important<br>2 Slightly Important<br>3 Moderately Important<br>4 Important<br>5 Very Important
24	descript_imp	A description of the condition (i.e. main physical features, gene(s) involved, etc.)	radio (Matrix)<br>1 Not Important<br>2 Slightly Important<br>3 Moderately Important<br>4 Important<br>5 Very Important
25	purpose_imp	The purpose of the session (i.e. reason for referral)	radio (Matrix)<br>1 Not Important<br>2 Slightly Important<br>3 Moderately Important<br>4 Important<br>5 Very Important
26	goal_imp	The goal of the session (i.e. to discuss genetic testing options, to give genetic testing results, etc.)	radio (Matrix)<br>1 Not Important<br>2 Slightly Important<br>3 Moderately Important<br>4 Important<br>5 Very Important
27	definition_imp	Definitions of important terminology	radio (Matrix)<br>1 Not Important<br>2 Slightly Important<br>3 Moderately Important<br>4 Important<br>5 Very Important
28	famdynam_imp	Family dynamics (i.e. child is adopted, parents are divorced, legal guardian is the grandmother, etc.)	radio (Matrix)<br>1 Not Important<br>2 Slightly Important<br>3 Moderately Important<br>4 Important<br>5 Very Important

29	empowering_patients	Section Header: *Please consider the following roles within a genetic counseling session. Please select for each role whether the role should be the responsibility of a genetic counselor (GC), a healthcare interpreter (HI), neither, or both.*<br>Empowering patients	radio (Matrix)<br>1 Always GC<br>2 Mostly GC<br>3 Equally GC and HI<br>4 Neither GC nor HI<br>5 Mostly HI<br>6 Always HI
30	clarifying_patient_underst	Clarifying patient understanding of information	radio (Matrix)<br>1 Always GC<br>2 Mostly GC<br>3 Equally GC and HI<br>4 Neither GC nor HI<br>5 Mostly HI<br>6 Always HI
31	improving_patient_health_l	Improving patient health literacy	radio (Matrix)<br>1 Always GC<br>2 Mostly GC<br>3 Equally GC and HI<br>4 Neither GC nor HI<br>5 Mostly HI<br>6 Always HI
32	advocating_for_the_patient	Advocating for the patient in the healthcare setting	radio (Matrix)<br>1 Always GC<br>2 Mostly GC<br>3 Equally GC and HI<br>4 Neither GC nor HI<br>5 Mostly HI<br>6 Always HI
33	assessing_patient_affect	Assessing patient affect	radio (Matrix)<br>1 Always GC<br>2 Mostly GC<br>3 Equally GC and HI<br>4 Neither GC nor HI<br>5 Mostly HI<br>6 Always HI
34	managing_patient_emotions	Managing patient emotions	radio (Matrix)<br>1 Always GC<br>2 Mostly GC<br>3 Equally GC and HI<br>4 Neither GC nor HI<br>5 Mostly HI<br>6 Always HI

35	expressing_empathy_verball	Expressing empathy verbally (word choice, empathy statements)	radio (Matrix)<br>1 Always GC<br>2 Mostly GC<br>3 Equally GC and HI<br>4 Neither GC nor HI<br>5 Mostly HI<br>6 Always HI
36	expressing_empathy_nonverb	Expressing empathy nonverbally (tone of voice, pacing)	radio (Matrix)<br>1 Always GC<br>2 Mostly GC<br>3 Equally GC and HI<br>4 Neither GC nor HI<br>5 Mostly HI<br>6 Always HI
37	engaging_in_relationship_b	Engaging in relationship-building with the patient	radio (Matrix)<br>1 Always GC<br>2 Mostly GC<br>3 Equally GC and HI<br>4 Neither GC nor HI<br>5 Mostly HI<br>6 Always HI
38	ensuring_cultural_approp	Ensuring the content is delivered in a culturally-appropriate way	radio (Matrix)<br>1 Always GC<br>2 Mostly GC<br>3 Equally GC and HI<br>4 Neither GC nor HI<br>5 Mostly HI<br>6 Always HI
39	maintaining_cultural_sensi	Maintaining understanding of patient's culture	radio (Matrix)<br>1 Always GC<br>2 Mostly GC<br>3 Equally GC and HI<br>4 Neither GC nor HI<br>5 Mostly HI<br>6 Always HI
40	responding_to_patient_cult	Reacting to patient cultural concerns	radio (Matrix)<br>1 Always GC<br>2 Mostly GC<br>3 Equally GC and HI<br>4 Neither GC nor HI<br>5 Mostly HI<br>6 Always HI

41	assessing_relevant_patient	Assessing for relevant patient cultural beliefs during the session	radio (Matrix)<br>1 Always GC<br>2 Mostly GC<br>3 Equally GC and HI<br>4 Neither GC nor HI<br>5 Mostly HI<br>6 Always HI
42	roleint_gc	Section Header:<br>Which title/description do you think best describes the role of a healthcare interpreter?	radio<br>1 Message converter - interpreter remains the voice of the patient and provider<br>2 Manager/Clarifier - interpreter checks for patient understanding and clarifies information to facilitate understanding<br>3 Cultural broker/liaison - interpreter has knowledge on the particular cultural beliefs of the individuals they are interpreting for, allowing the interpreter to detect cultural misunderstandings and provide the necessary cultural framework to clear up any misunderstandings<br>4 Patient advocate - interpreter acts on certain issues if they feel that the patient's health, well-being, or dignity is at risk<br>5 Other: {roleintbox_gc}<br><br>Custom alignment: LV
43	roleintbox_gc		text
44	resources_gc	Section Header:<br>Have you ever provided resources regarding interpreting in genetics to interpreters you worked with?	yesno<br>1 Yes<br>0 No
45	seminars_webinars_gc<br>Show the field ONLY if: [resources_gc] = '1'	Section Header: *How often do you provide the following resources regarding interpreting in genetics to an interpreter?*<br>Seminars/Webinars hosted by genetic counselors	radio (Matrix)<br>1 Never<br>2 Rarely<br>3 Sometimes<br>4 Usually<br>5 Always
46	online_genetics_resources<br>Show the field ONLY if: [resources_gc] = '1'	Online genetics resources (i.e. Genetics Home Reference, Genereviews)	radio (Matrix)<br>1 Never<br>2 Rarely<br>3 Sometimes<br>4 Usually<br>5 Always
47	list_genetic_terms_gc<br>Show the field ONLY if: [resources_gc] = '1'	List of common genetic terminology	radio (Matrix)<br>1 Never<br>2 Rarely<br>3 Sometimes<br>4 Usually<br>5 Always

48	online_medical_genetics_gc<br>Show the field ONLY if:<br>[resources_gc] = '1'	Online medical genetics translation tool (i.e. Lexigene)	radio (Matrix)<br>1 Never<br>2 Rarely<br>3 Sometimes<br>4 Usually<br>5 Always
49	patient_resource_gc<br>Show the field ONLY if:<br>[resources_gc] = '1'	Patient resource pamphlets	radio (Matrix)<br>1 Never<br>2 Rarely<br>3 Sometimes<br>4 Usually<br>5 Always
50	resourcesadd_gc<br>Show the field ONLY if:<br>[resources_gc] = 1	Please describe any resources not listed above that you have provided to healthcare interpreters (if applicable).	notes<br>Custom alignment: LV
51	resourcesinit_gc<br>Show the field ONLY if:<br>[resources_gc] = '1'	When you provided these resources, who initiated the conversation?	radio<br>1 I, the genetic counselor, did.<br>2 The healthcare interpreter did.<br>3 I have both initiated and had a healthcare interpreter initiate this conversation.
52	lacktime_gc	Section Header: *How often do you encounter the following constraints when working with interpreters:*<br>Lack of time for patient session	radio (Matrix)<br>1 Never<br>2 Rarely<br>3 Sometimes<br>4 Usually<br>5 Always<br>6 Not applicable
53	lackfamiliarity_gc	Interpreter lack of familiarity with genetic terminology	radio (Matrix)<br>1 Never<br>2 Rarely<br>3 Sometimes<br>4 Usually<br>5 Always<br>6 Not applicable
54	lackcomm_gc	Lack of communication from interpreter (pre-session)	radio (Matrix)<br>1 Never<br>2 Rarely<br>3 Sometimes<br>4 Usually<br>5 Always<br>6 Not applicable
55	lacktrust_gc	Lack of trust in interpretations made by interpreter	radio (Matrix)<br>1 Never<br>2 Rarely<br>3 Sometimes<br>4 Usually<br>5 Always<br>6 Not applicable

56	clarquest_gc	Feeling unable to ask clarifying questions of interpreter	radio (Matrix)<br>1 Never<br>2 Rarely<br>3 Sometimes<br>4 Usually<br>5 Always<br>6 Not applicable
57	respect_gc	Not feeling respected during a session	radio (Matrix)<br>1 Never<br>2 Rarely<br>3 Sometimes<br>4 Usually<br>5 Always<br>6 Not applicable
58	techissue_gc	Technology issues (if working with remote interpreter)	radio (Matrix)<br>1 Never<br>2 Rarely<br>3 Sometimes<br>4 Usually<br>5 Always<br>6 Not applicable
59	sched_gc	Insufficient time provided by department schedule to meet session needs	radio (Matrix)<br>1 Never<br>2 Rarely<br>3 Sometimes<br>4 Usually<br>5 Always<br>6 Not applicable
60	lacktraining_gc	Lack of training in working with interpreters	radio (Matrix)<br>1 Never<br>2 Rarely<br>3 Sometimes<br>4 Usually<br>5 Always<br>6 Not applicable
61	constraintsother_gc	Please describe constraints you have encountered while working with interpreters that are not listed above and how frequently you encounter them (if applicable).	notes<br>Custom alignment: LV
62	relationshipimp_gc	Section Header:<br>What suggestions do you have on how the genetic counselor and healthcare interpreter working relationship can be improved?	notes<br>Custom alignment: LV
63	demo_age	Section Header: *The main portion of the survey is now complete. The following questions will ask about demographic information.*<br>What is your current age in years (round to the nearest year)?	text (integer, Min: 18, Max: 100)

64	demo_gender	What is your gender?	radio<br>1 Male<br>2 Female<br>3 Transgender<br>4 Non-Binary<br>5 Gender Fluid<br>6 Other (please specify): {demo_genderbox}<br>7 Prefer not to say
65	demo_genderbox		text
66	education_gc	What is the highest degree you have completed?	radio<br>1 Master's Degree (i.e. MA, MS)<br>2 Professional Degree (i.e. MD, DDS, JD)<br>3 Doctorate (i.e. PhD, EdD)
67	demo_hisp	Do you identify as Hispanic or Latino?	yesno<br>1 Yes<br>0 No
68	demo_raceeth	How would you identify your race/ethnicity (select all that apply)?	checkbox<br>1 demo_raceeth___1 American Indian or Alaskan Native<br>2 demo_raceeth___2 Asian<br>3 demo_raceeth___3 South Asian<br>4 demo_raceeth___4 Black or African American<br>5 demo_raceeth___5 Native Hawaiian or Other Pacific Islander<br>6 demo_raceeth___6 White<br>7 demo_raceeth___7 Other (Please specify): {demoraceethbox}<br>8 demo_raceeth___8 I would rather not specify<br><br>Field Annotation: @NONEOFTHEABOVE = 8
69	demoraceethbox		text
70	gc_survey_complete	Section Header: *Form Status*<br>Complete?	dropdown<br>0 Incomplete<br>1 Unverified<br>2 Complete
