# Supplementary material for: Exploring United States genetic counselor and healthcare interpreter perspectives: Allocation of roles within the genetic counseling encounter
Source: J Genet Couns. 2022 Apr 13;31(4):976–88. doi: 10.1002/jgc4.1572 (PMC9542924; doi:10.1002/jgc4.1572)
Supplement: Supplementary file 3 — Data S3 [file JGC4-31-976-s006.pdf]

Genetic Counselor and Interpreter Perspectives: Allocation of Roles - Interpreter Survey PID 5804

Codebook

Data Dictionary Codebook

12/17/2020 6:11pm

Collapse all instruments

#	Variable / Field Name	Field Label<br>*Field Note*	Field Attributes (Field Type, Validation, Choices, Calculations, etc.)
Instrument: **Interpreter Survey** (interpreter_survey) Enabled as survey			Collapse
1	record_id	Record ID	text
2	consent_int	By selecting "I Agree", you understand the information above and agree to the terms of this study.	radio, Required<br>1 I Agree<br>Custom alignment: LV
3	screen_age_int	Section Header:<br>Are you 18 years of age or older?	yesno, Required<br>1 Yes<br>0 No<br>Custom alignment: LV<br>Stop actions on 0
4	screen_practice_int	Section Header:<br>Have you practiced as a healthcare interpreter in the United States within the last 5 calendar years?	yesno, Required<br>1 Yes<br>0 No<br>Custom alignment: LV<br>Stop actions on 0
5	screen_workgc_int	Section Header:<br>Have you worked with a genetic counselor before? Definition from the National Society of Genetic Counselors: "Genetic counselors are professionals who have advanced training in medical genetics and counseling to interpret genetic test results and to guide and support patients seeking more information about such things as: How inherited diseases and conditions might affect them or their families. How family and medical histories may impact the chance of disease occurrence or recurrence. Which genetic tests may or may not be right for them, and what those tests may or may not tell. How to make the most informed choices about healthcare conditions. Most genetic counselors work in a clinic or hospital and often work with obstetricians, oncologists and other doctors. Like doctors, genetic counselors can work in a variety of settings and provide different services."	radio, Required<br>1 Yes<br>0 No<br>2 Unsure<br>Custom alignment: LV
6	screen_yearspractice_int	Section Header:<br>How many years have you been a practicing healthcare interpreter in the United States (round to the nearest year; if you have been a healthcare interpreter for less than one year, please round up to one)?	text (integer, Min: 1, Max: 100)

7	screen_timesgc_int<br>Show the field ONLY if:<br>[screen_workgc_int] = 1	Thinking about from the start of your career working as a healthcare interpreter, approximately how many times have you worked with a genetic counselor?	dropdown<br>1 1 time<br>2 2 times<br>3 3-5 times<br>4 6-10 times<br>5 11-20 times<br>6 21-30 times<br>7 31-40 times<br>8 41-50 times<br>9 51-60 times<br>10 61-70 times<br>11 71-80 times<br>12 81-90 times<br>13 91-100 times<br>14 101+ times
8	specialtiesgc_int<br>Show the field ONLY if:<br>[screen_workgc_int] = 1	Which genetic counseling specialties have you interpreted for (select all that apply)?	checkbox<br>1 specialtiesgc_int___1 Pediatrics - childhood onset conditions, newborns in the NICU/PICU<br>2 specialtiesgc_int___2 Prenatal - pregnant women, discussion of pregnancy screening and testing options<br>3 specialtiesgc_int___3 Cancer - pediatric or adult<br>4 specialtiesgc_int___4 Adult (non-cancer) - cardiology, neurology, etc.<br>5 specialtiesgc_int___5 Other (please specify): {specialtiesgcbox_int}<br>6 specialtiesgc_int___6 Unknown - I have worked with a genetic counselor but I am unsure in what specialty.
9	specialtiesgcbox_int<br>Show the field ONLY if:<br>[screen_workgc_int] = 1		text

10	regions_int	Section Header:<br>In which regions of the United States have you provided healthcare interpreting services (select all that apply)?	checkbox<br>1 regions_int___1 Region 1 - New England (Connecticut, Maine, Massachusetts, New Hampshire, Rhode Island, Vermont)<br>2 regions_int___2 Region 2 - Middle Atlantic (New Jersey, New York, Pennsylvania)<br>3 regions_int___3 Region 3 - South Atlantic (Delaware, District of Columbia, Florida, Georgia, Maryland, North Carolina, South Carolina, Virginia, West Virginia)<br>4 regions_int___4 Region 4 - North Central (Kansas, Illinois, Indiana, Iowa, Michigan, Minnesota, Missouri, Nebraska, North Dakota, Ohio, South Dakota, Wisconsin)<br>5 regions_int___5 Region 5 - South Central (Alabama, Arkansas, Kentucky, Louisiana, Mississippi, Oklahoma, Tennessee, Texas)<br>6 regions_int___6 Region 6 - Mountain (Arizona, Colorado, Idaho, Montana, Nevada, New Mexico, Utah, Wyoming)<br>7 regions_int___7 Region 7 - West (Alaska, California, Hawaii, Oregon, Washington)<br>8 regions_int___8 I provide services across several states remotely.<br><br>Custom alignment: LV
11	language_gc	Section Header:<br>For what language(s), in addition to English, do you provide healthcare interpreting services (select all the apply)?	checkbox<br>1 language_gc___1 American Sign Language<br>40 language_gc___40 Amharic, Somali, or other Afro-Asiatic languages<br>2 language_gc___2 Arabic<br>3 language_gc___3 Armenian<br>4 language_gc___4 Bengali<br>5 language_gc___5 Chinese (incl. Mandarin, Cantonese)<br>6 language_gc___6 French (incl. Cajun)<br>7 language_gc___7 German<br>8 language_gc___8 Greek<br>9 language_gc___9 Gujarati<br>10 language_gc___10 Haitian<br>11 language_gc___11 Hebrew<br>12 language_gc___12 Hindi<br>13 language_gc___13 Hmong<br>34 language_gc___34 Ilocano, Samoan, Hawaiian, or other Austronesian languages<br>14 language_gc___14 Italian<br>15 language_gc___15 Japanese<br>16 language_gc___16 Khmer<br>17 language_gc___17 Korean

<table>
<tr><td></td><td></td><td></td><td>
<table>
<tr><td>35</td><td>language_gc___35</td><td>Malayalam, Kannada, or other Dravidian languages</td></tr>
<tr><td>18</td><td>language_gc___18</td><td>Navajo</td></tr>
<tr><td>36</td><td>language_gc___36</td><td>Nepali, Marathi, or other Indic languages</td></tr>
<tr><td>19</td><td>language_gc___19</td><td>Persian (incl. Farsi, Dari)</td></tr>
<tr><td>20</td><td>language_gc___20</td><td>Polish</td></tr>
<tr><td>21</td><td>language_gc___21</td><td>Portuguese</td></tr>
<tr><td>22</td><td>language_gc___22</td><td>Punjabi</td></tr>
<tr><td>23</td><td>language_gc___23</td><td>Russian</td></tr>
<tr><td>24</td><td>language_gc___24</td><td>Serbo-Croatian</td></tr>
<tr><td>25</td><td>language_gc___25</td><td>Spanish</td></tr>
<tr><td>26</td><td>language_gc___26</td><td>Swahili or other languages of Central, Eastern, and Southern Africa</td></tr>
<tr><td>27</td><td>language_gc___27</td><td>Tagalog (incl. Filipino)</td></tr>
<tr><td>28</td><td>language_gc___28</td><td>Tamil</td></tr>
<tr><td>29</td><td>language_gc___29</td><td>Telugu</td></tr>
<tr><td>37</td><td>language_gc___37</td><td>Thai, Lao, or other Tai-Kadai languages</td></tr>
<tr><td>30</td><td>language_gc___30</td><td>Ukrainian or other Slavic languages</td></tr>
<tr><td>31</td><td>language_gc___31</td><td>Urdu</td></tr>
<tr><td>32</td><td>language_gc___32</td><td>Vietnamese</td></tr>
<tr><td>38</td><td>language_gc___38</td><td>Yiddish, Pennsylvania Dutch or other West Germanic languages</td></tr>
<tr><td>39</td><td>language_gc___39</td><td>Yoruba, Twi, Igbo, or other languages of Western Africa</td></tr>
<tr><td>33</td><td>language_gc___33</td><td>Other (please specify): {languagebox_int}</td></tr>
</table>
Custom alignment: LV</td></tr>
<tr><td>12</td><td>languagebox_int</td><td></td><td>text</td></tr>
<tr><td>13</td><td>cert_int</td><td>Section Header:
Do you have certification for interpreting in healthcare?</td><td>yesno
<table>
<tr><td>1</td><td>Yes</td></tr>
<tr><td>0</td><td>No</td></tr>
</table>
Custom alignment: LV</td></tr>
<tr><td>14</td><td>certwork_int
Show the field ONLY if: [cert_int] = 0</td><td>Section Header:
Are you working towards certification for interpreting in healthcare?</td><td>yesno
<table>
<tr><td>1</td><td>Yes</td></tr>
<tr><td>0</td><td>No</td></tr>
</table>
Custom alignment: LV</td></tr>
<tr><td>15</td><td>certorg_int
Show the field ONLY if: [certwork_int] = 1 or [cert_int] = 1</td><td>Section Header:
Through which organization did you receive or are you working towards certification for interpreting in healthcare?</td><td>radio
<table>
<tr><td>1</td><td>National Board of Certification for Medical Interpreters (NBCMI)</td></tr>
<tr><td>2</td><td>Certification Commission for Healthcare Interpreters (CCHI)</td></tr>
<tr><td>3</td><td>Other certification body (please specify): {certorgbox_int}</td></tr>
</table>
Custom alignment: LV</td></tr>
</table>

16	certorgbox_int<br><br>Show the field ONLY if:<br>[certwork_int] = 1 or [cert_int] =1		text
17	casescenario_int<br><br>Show the field ONLY if:<br>[screen_workgc_int] = 0 or [screen_workgc_int] = 2	Section Header:<br>Please read the following case scenario: You are scheduled to interpret for a family with a 1 month-old baby with Down syndrome. The family has limited English proficiency and speaks the language you interpret for. They are coming to the visit to receive the genetic testing results confirming the baby's diagnosis of Down syndrome. The remaining questions will pertain to the case scenario. Please answer the following questions in consideration of how you might respond if you were presented with this case.	radio<br>1 I have read the case scenario.<br><br>Custom alignment: LV
18	methodsgc_int<br><br>Show the field ONLY if:<br>[screen_workgc_int] = 1	Section Header:<br>Through what methods have you provided interpreting services for a genetic counseling session (select all that apply)?	checkbox<br>1 methodsgc_int___1 In-person<br>2 methodsgc_int___2 Telephone through hospital (in-house)<br>3 methodsgc_int___3 Telehealth (with audio and video) through hospital (in-house)<br>4 methodsgc_int___4 Telephone through outside agency<br>5 methodsgc_int___5 Telehealth (with audio and video) through outside agency<br>6 methodsgc_int___6 Other (please specify): {methodsgcbox_int}<br><br>Custom alignment: LV
19	methodsgcbox_int<br><br>Show the field ONLY if:<br>[screen_workgc_int] = 1		text
20	methodsgcpref_int<br><br>Show the field ONLY if:<br>[screen_workgc_int] = 1	Of the methods you have provided interpreting services in the past, what is your preferred method of providing interpreting services for a genetic counseling session?	radio<br>1 In-person<br>2 Telephone through hospital (in-house)<br>3 Telehealth (with audio and video) through hospital (in-house)<br>4 Telephone through outside agency<br>5 Telehealth (with audio and video) through outside agency<br>6 Other (please specify): {methodsgcprefbox_int}<br><br>Custom alignment: LV
21	methodsgcprefbox_int<br><br>Show the field ONLY if:<br>[screen_workgc_int] = 1		text
22	name_int<br><br>Show the field ONLY if:<br>[screen_workgc_int] = 1	Section Header: *How often does a genetic counselor discuss with you before the session the following topics:*<br>The name of the condition the patient has or is at risk for	radio (Matrix)<br>1 Never<br>2 Rarely<br>3 Sometimes<br>4 Often<br>5 Always

23	descript_int<br>Show the field ONLY if:<br>[screen_workgc_int] = 1	A description of the condition (i.e. main physical features, gene(s) involved, etc.)	radio (Matrix)<br>1 Never<br>2 Rarely<br>3 Sometimes<br>4 Often<br>5 Always
24	purpose_int<br>Show the field ONLY if:<br>[screen_workgc_int] = 1	The purpose of the session (i.e. reason for referral)	radio (Matrix)<br>1 Never<br>2 Rarely<br>3 Sometimes<br>4 Often<br>5 Always
25	goal_int<br>Show the field ONLY if:<br>[screen_workgc_int] = 1	The goal of the session (i.e. to discuss genetic testing options, to give genetic testing results, etc.)	radio (Matrix)<br>1 Never<br>2 Rarely<br>3 Sometimes<br>4 Often<br>5 Always
26	definition_int<br>Show the field ONLY if:<br>[screen_workgc_int] = 1	Definitions of important terminology	radio (Matrix)<br>1 Never<br>2 Rarely<br>3 Sometimes<br>4 Often<br>5 Always
27	famdynam_int<br>Show the field ONLY if:<br>[screen_workgc_int] = 1	Family dynamics (i.e. child is adopted, parents are divorced, legal guardian is the grandmother, etc.)	radio (Matrix)<br>1 Never<br>2 Rarely<br>3 Sometimes<br>4 Often<br>5 Always
28	name_imp	Section Header: *How important is it that a genetic counselor discusses with you before the session the following topics:*<br>The name of the condition the patient has or is at risk for	radio (Matrix)<br>1 Not Important<br>2 Slightly Important<br>3 Moderately Important<br>4 Important<br>5 Very Important
29	descript_imp	A description of the condition (i.e. main physical features, gene(s) involved, etc.)	radio (Matrix)<br>1 Not Important<br>2 Slightly Important<br>3 Moderately Important<br>4 Important<br>5 Very Important
30	purpose_imp	The purpose of the session (i.e. reason for referral)	radio (Matrix)<br>1 Not Important<br>2 Slightly Important<br>3 Moderately Important<br>4 Important<br>5 Very Important

31	goal_imp	The goal of the session (i.e. to discuss genetic testing options, to give genetic testing results, etc.)	radio (Matrix)<br>1 Not Important<br>2 Slightly Important<br>3 Moderately Important<br>4 Important<br>5 Very Important
32	definition_imp	Definitions of important terminology	radio (Matrix)<br>1 Not Important<br>2 Slightly Important<br>3 Moderately Important<br>4 Important<br>5 Very Important
33	famdynam_imp	Family dynamics (i.e. child is adopted, parents are divorced, legal guardian is the grandmother, etc.)	radio (Matrix)<br>1 Not Important<br>2 Slightly Important<br>3 Moderately Important<br>4 Important<br>5 Very Important
34	empowering_patients	Section Header: *Please consider the following roles during a genetic counseling session. Please select for each role whether the role should be the responsibility of a genetic counselor (GC), a healthcare interpreter (HI), neither, or both.*<br>Empowering patients	radio (Matrix)<br>1 Always GC<br>2 Mostly GC<br>3 Equally GC and HI<br>4 Neither GC nor HI<br>5 Mostly HI<br>6 Always HI
35	clarifying_patient_underst	Clarifying patient understanding of information	radio (Matrix)<br>1 Always GC<br>2 Mostly GC<br>3 Equally GC and HI<br>4 Neither GC nor HI<br>5 Mostly HI<br>6 Always HI
36	improving_patient_health_l	Improving patient health literacy	radio (Matrix)<br>1 Always GC<br>2 Mostly GC<br>3 Equally GC and HI<br>4 Neither GC nor HI<br>5 Mostly HI<br>6 Always HI
37	advocating_for_the_patient	Advocating for the patient in the healthcare setting	radio (Matrix)<br>1 Always GC<br>2 Mostly GC<br>3 Equally GC and HI<br>4 Neither GC nor HI<br>5 Mostly HI<br>6 Always HI

38	assessing_patient_affect	Assessing patient affect	radio (Matrix)<br>1 Always GC<br>2 Mostly GC<br>3 Equally GC and HI<br>4 Neither GC nor HI<br>5 Mostly HI<br>6 Always HI
39	managing_patient_emotions	Managing patient emotions	radio (Matrix)<br>1 Always GC<br>2 Mostly GC<br>3 Equally GC and HI<br>4 Neither GC nor HI<br>5 Mostly HI<br>6 Always HI
40	expressing_empathy_verball	Expressing empathy verbally (word choice, empathy statements)	radio (Matrix)<br>1 Always GC<br>2 Mostly GC<br>3 Equally GC and HI<br>4 Neither GC nor HI<br>5 Mostly HI<br>6 Always HI
41	expressing_empathy_nonverb	Expressing empathy nonverbally (tone of voice, pacing)	radio (Matrix)<br>1 Always GC<br>2 Mostly GC<br>3 Equally GC and HI<br>4 Neither GC nor HI<br>5 Mostly HI<br>6 Always HI
42	engaging_in_relationship_b	Engaging in relationship-building with the patient	radio (Matrix)<br>1 Always GC<br>2 Mostly GC<br>3 Equally GC and HI<br>4 Neither GC nor HI<br>5 Mostly HI<br>6 Always HI
43	ensuring_cultural_approp	Ensuring the content is delivered in a culturally-appropriate way	radio (Matrix)<br>1 Always GC<br>2 Mostly GC<br>3 Equally GC and HI<br>4 Neither GC nor HI<br>5 Mostly HI<br>6 Always HI

44	maintaining_cultural_sensi	Maintaining understanding of patient's culture	radio (Matrix)<br>1 Always GC<br>2 Mostly GC<br>3 Equally GC and HI<br>4 Neither GC nor HI<br>5 Mostly HI<br>6 Always HI
45	responding_to_patient_cult	Reacting to patient cultural concerns	radio (Matrix)<br>1 Always GC<br>2 Mostly GC<br>3 Equally GC and HI<br>4 Neither GC nor HI<br>5 Mostly HI<br>6 Always HI
46	assessing_relevant_patient	Assessing relevant patient cultural beliefs for the session	radio (Matrix)<br>1 Always GC<br>2 Mostly GC<br>3 Equally GC and HI<br>4 Neither GC nor HI<br>5 Mostly HI<br>6 Always HI
47	roleint_int	Section Header:<br>Which title/description do you think best describes the role of a healthcare interpreter?	radio<br>1 Message converter - interpreter remains the voice of the patient and provider<br>2 Manager/Clarifier - interpreter checks for patient understanding and clarifies information to facilitate understanding<br>3 Cultural broker/liaison - interpreter has knowledge on the particular cultural beliefs of the individuals they are interpreting for, allowing the interpreter to detect cultural misunderstandings and provide the necessary cultural framework to clear up any misunderstandings<br>4 Patient advocate - interpreter acts on certain issues if they feel that the patient's health, well-being, or dignity is at risk<br>5 Other: {roleintbox_int}<br><br>Custom alignment: LV
48	roleintbox_int		text
49	resources_int<br>Show the field ONLY if:<br>[screen_workgc_int] = 1	Section Header:<br>Have you received resources related to interpreting in genetic counseling from a genetic counselor you worked with?	yesno<br>1 Yes<br>0 No<br><br>Custom alignment: LV

50	resourcestypes_int<br>Show the field ONLY if:<br>[resources_int] = 1 and [screen_workgc_int] = 1	Section Header:<br>Which of the following resources have you received from genetic counselors (select all that apply)?	checkbox<br>1 resourcestypes_int___1 Seminars/Webinars hosted by genetic counselors<br>2 resourcestypes_int___2 Online genetics resources (i.e. Genetics Home Reference, Genereviews)<br>3 resourcestypes_int___3 List of common genetic terminology<br>4 resourcestypes_int___4 Online medical genetics translation tool (i.e. Lexigene)<br>5 resourcestypes_int___5 Patient resource pamphlets<br>6 resourcestypes_int___6 Other (please specify): {resourcestypesbox_int}<br>Custom alignment: LV
51	resourcestypesbox_int<br>Show the field ONLY if:<br>[resources_int] = 1 and [screen_workgc_int] = 1		text
52	resourcesinit_int<br>Show the field ONLY if:<br>[resources_int] = '1' and [screen_workgc_int] = 1	When you received these resources, who initiated the conversation?	radio<br>1 I, the healthcare interpreter, did.<br>2 The genetic counselor did.<br>3 I have both initiated and had a genetic counselor initiate this conversation.<br>Custom alignment: LV
53	resources_int2<br>Show the field ONLY if:<br>[screen_workgc_int] = 0 or [screen_workgc_int] = 2 or [resources_int] = 0	Section Header:<br>Have you ever accessed resources related to interpreting in genetic counseling?	yesno<br>1 Yes<br>0 No<br>Custom alignment: LV
54	resourcestypes_int2<br>Show the field ONLY if:<br>[resources_int2] = 1	Section Header:<br>What resources have you accessed (select all that apply)?	checkbox<br>1 resourcestypes_int2___1 Seminars/Webinars hosted by genetic counselors<br>2 resourcestypes_int2___2 Online genetics resources (i.e. Genetics Home Reference, Genereviews)<br>3 resourcestypes_int2___3 List of common genetic terminology<br>4 resourcestypes_int2___4 Online medical genetics translation tool (i.e. Lexigene)<br>5 resourcestypes_int2___5 Patient resource pamphlets<br>6 resourcestypes_int2___6 Other (please specify): {resourcestypesbox_int2}<br>Custom alignment: LV
55	resourcestypesbox_int2<br>Show the field ONLY if:<br>[resources_int2] = 1		text

56	resourcesgc_int<br><br>Show the field ONLY if:<br>[screen_workgc_int] = 0 or [screen_workgc_int] = 2 or [resources_int] = 0	Section Header:<br>Would you like to receive resources related to interpreting in genetic counseling from a genetic counselor?	yesno<br>1 Yes<br>0 No<br><br>Custom alignment: LV
57	resourceshelp_int<br><br>Show the field ONLY if:<br>[resourcesgc_int] = 1 or [resources_int] = 1	Section Header:<br>What resources would be helpful to receive from a genetic counselor (select all that apply)?	checkbox<br>1 resourceshelp_int___1 Seminars/Webinars hosted by genetic counselors<br>2 resourceshelp_int___2 Online genetics resources (i.e. Genetics Home Reference, Genereviews)<br>3 resourceshelp_int___3 List of common genetic terminology<br>4 resourceshelp_int___4 Online medical genetics translation tool (i.e. Lexigene)<br>5 resourceshelp_int___5 Patient resource pamphlets<br>6 resourceshelp_int___6 Other (please specify): {resourceshelpbox_int}<br><br>Custom alignment: LV
58	resourceshelpbox_int		text
59	lacktime_int<br><br>Show the field ONLY if:<br>[screen_workgc_int] = 1	Section Header: *How often do you encounter the following constraints when working with genetic counselors?*<br>Lack of time for patient session	radio (Matrix)<br>1 Never<br>2 Rarely<br>3 Sometimes<br>4 Usually<br>5 Always<br>6 Not applicable
60	lackfamiliarity_int<br><br>Show the field ONLY if:<br>[screen_workgc_int] = 1	Lack of familiarity with genetic terminology	radio (Matrix)<br>1 Never<br>2 Rarely<br>3 Sometimes<br>4 Usually<br>5 Always<br>6 Not applicable
61	infofromgc_int<br><br>Show the field ONLY if:<br>[screen_workgc_int] = 1	Insufficient information pre-session from genetic counselor to provide interpreting	radio (Matrix)<br>1 Never<br>2 Rarely<br>3 Sometimes<br>4 Usually<br>5 Always<br>6 Not applicable
62	lacktrust_int<br><br>Show the field ONLY if:<br>[screen_workgc_int] = 1	Lack of trust from genetic counselor of my interpreting	radio (Matrix)<br>1 Never<br>2 Rarely<br>3 Sometimes<br>4 Usually<br>5 Always<br>6 Not applicable

63	clarquest_int<br>Show the field ONLY if:<br>[screen_workgc_int] = 1	Feeling unable to ask clarifying questions of genetic counselor	radio (Matrix)<br>1 Never<br>2 Rarely<br>3 Sometimes<br>4 Usually<br>5 Always<br>6 Not applicable
64	respect_int<br>Show the field ONLY if:<br>[screen_workgc_int] = 1	Not feeling respected during a session	radio (Matrix)<br>1 Never<br>2 Rarely<br>3 Sometimes<br>4 Usually<br>5 Always<br>6 Not applicable
65	techissue_int<br>Show the field ONLY if:<br>[screen_workgc_int] = 1	Technology issues (if working remotely)	radio (Matrix)<br>1 Never<br>2 Rarely<br>3 Sometimes<br>4 Usually<br>5 Always<br>6 Not applicable
66	sched_int<br>Show the field ONLY if:<br>[screen_workgc_int] = 1	Insufficient time provided by department schedule to meet session needs	radio (Matrix)<br>1 Never<br>2 Rarely<br>3 Sometimes<br>4 Usually<br>5 Always<br>6 Not applicable
67	lacktraining_int<br>Show the field ONLY if:<br>[screen_workgc_int] = 1	Lack of training in interpreting in genetics	radio (Matrix)<br>1 Never<br>2 Rarely<br>3 Sometimes<br>4 Usually<br>5 Always<br>6 Not applicable
68	lacktraininggc_int<br>Show the field ONLY if:<br>[screen_workgc_int] = 1	Lack of training in interpreting in genetic counseling	radio (Matrix)<br>1 Never<br>2 Rarely<br>3 Sometimes<br>4 Usually<br>5 Always<br>6 Not applicable
69	constraintsother_int<br>Show the field ONLY if:<br>[screen_workgc_int] = 1	Please describe constraints you have encountered while collaborating with genetic counselors that are not listed above and how frequently you encounter them (if applicable).	notes<br>Custom alignment: LV
70	relationshipimp_int<br>Show the field ONLY if:<br>[screen_workgc_int] = 1	Section Header:<br>What suggestions do you have on how the genetic counselor and healthcare interpreter relationship can be improved?	notes<br>Custom alignment: LV

71	demo_age	Section Header: *The main portion of the survey is now complete. The following questions will ask about demographic information.*<br>What is your current age in years (round to the nearest year)?	text (integer, Min: 18, Max: 100)
72	education_int	What is the highest degree or level of school you have completed?	radio<br>1 Less than high school diploma<br>2 High school diploma<br>3 Some college, but no degree<br>4 Associates Degree (i.e. AA, AS)<br>5 Bachelor's Degree (i.e. BA, BS)<br>6 Master's Degree (i.e. MA, MS)<br>7 Professional Degree (i.e. MD, DDS, JD)<br>8 Doctorate (i.e. PhD, EdD)
73	demo_gender	What is your gender?	radio<br>1 Male<br>2 Female<br>3 Transgender<br>4 Non-Binary<br>5 Gender Fluid<br>6 Other (please specify): {demo_genderbox}<br>7 Prefer not to say
74	demo_genderbox		text
75	demo_hisp	Do you identify as Hispanic or Latino?	yesno<br>1 Yes<br>0 No
76	demo_raceeth	How would you identify your race/ethnicity (select all that apply)?	checkbox<br>1 demo_raceeth___1 American Indian or Alaskan Native<br>2 demo_raceeth___2 Asian<br>3 demo_raceeth___3 South Asian<br>4 demo_raceeth___4 Black or African American<br>5 demo_raceeth___5 Native Hawaiian or Other Pacific Islander<br>6 demo_raceeth___6 White<br>7 demo_raceeth___7 Other (Please specify): {demoraceethbox}<br>8 demo_raceeth___8 I would rather not specify<br><br>Field Annotation: @NONEOFTHEABOVE = 8
77	demoraceethbox		text
78	interpreter_survey_complete	Section Header: *Form Status*<br>Complete?	dropdown<br>0 Incomplete<br>1 Unverified<br>2 Complete
