# Supplementary material for: Exploring United States genetic counselor and healthcare interpreter perspectives: Allocation of roles within the genetic counseling encounter
Source: J Genet Couns. 2022 Apr 13;31(4):976–88. doi: 10.1002/jgc4.1572 (PMC9542924; doi:10.1002/jgc4.1572)
Supplement: Supplementary file 4 — Data S4 [file JGC4-31-976-s003.docx]

**Supplemental Data 4.** Description of Genetic Counselors and Case Scenario Provided to Healthcare Interpreters

**Description of Genetic Counselors:**

"Genetic counselors are professionals who have advanced training in medical genetics and counseling to interpret genetic test results and to guide and support patients seeking more information about such things as:

- How inherited diseases and conditions might affect them or their families.
- How family and medical histories may impact the chance of disease occurrence or recurrence.
- Which genetic tests may or may not be right for them, and what those tests may or may not tell.
- How to make the most informed choices about healthcare conditions.

Most genetic counselors work in a clinic or hospital and often work with obstetricians, oncologists and other doctors. Like doctors, genetic counselors can work in a variety of settings and provide different services.”

**Case Scenario:**

“You are scheduled to interpret for a family with a 1 month-old baby with Down syndrome. The family has limited English proficiency and speaks the language you interpret for. They are coming to the visit to receive the genetic testing results confirming the baby's diagnosis of Down syndrome.”
