# Supplementary material for: Exploring United States genetic counselor and healthcare interpreter perspectives: Allocation of roles within the genetic counseling encounter
Source: J Genet Couns. 2022 Apr 13;31(4):976–88. doi: 10.1002/jgc4.1572 (PMC9542924; doi:10.1002/jgc4.1572)
Supplement: Supplementary file 5 — Data S5 [file JGC4-31-976-s007.docx]

**Supplemental Data 5.** Methods Previously Utilized and Preferred by Genetic Counselors and Healthcare Interpreters to Receive or Provide Interpreting Services for a Genetic Counseling Session

| **Method** | **Genetic Counselor** | | **Healthcare Interpreter** | |
| --- | --- | --- | --- | --- |
|  | **Previously Utilized (participants could select >1)**  **(n=130) (%)** | **Preferred^a^**  **(n=125) (%)** | **Previously Utilized (participants could select >1)**  **(n=29) (%)** | **Preferred^b^**  **(n=9) (%)** |
| **In-person** | 122 (93.8) | 110 (88.0) | 29 (100.0) | 7 (77.8) |
| **Telephone (In-house)** | 65 (50.0) | 3 (2.4) | 6 (20.7) | 1 (11.1) |
| **Telehealth (In-house)** | 37 (28.5) | 1 (0.8) | 3 (10.3) | 1 (11.1) |
| **Telephone (Outside Agency)** | 108 (83.1) | 3 (2.4) | 3 (10.3) | 0 (0.0) |
| **Telehealth (Outside Agency)** | 66 (50.8) | 7 (5.6) | 2(6.9) | 0 (0.0) |
| **Other** | 2 (1.5) | 0 (0.0) | 0 (0.0) | 0 (0.0) |

1. 5/130 (3.8%) GCs have previously only utilized one method (3 in-person only, 2 telephone outside agency only) to receive interpreting services for a genetic counseling session and were omitted.
2. 19/29 (65.5%) HIs have previously only utilized one method (19 in-person only) to provide interpreting services for a genetic counseling session and were omitted. One participant did not respond.
