# Supplementary material for: Exploring United States genetic counselor and healthcare interpreter perspectives: Allocation of roles within the genetic counseling encounter
Source: J Genet Couns. 2022 Apr 13;31(4):976–88. doi: 10.1002/jgc4.1572 (PMC9542924; doi:10.1002/jgc4.1572)
Supplement: Supplementary file 6 — Data S6‐S7 [file JGC4-31-976-s002.docx]

**Supplemental Data 6.** Mann-U Whitney Outputs Comparing Genetic Counselor and Healthcare Interpreter Perspectives on Topics During a Pre-Session

1. Frequency

|  | Name of Condition | Description of Condition | Purpose of Session | Goal of Session | Definition of Important Genetic Terminology | Family Dynamics |
| --- | --- | --- | --- | --- | --- | --- |
| Mann-Whitney U | 1385.000 | 1289.500 | 1739.000 | 1592.500 | 1511.000 | 1634.000 |
| Wilcoxon W | 9900.000 | 9804.500 | 10254.000 | 10107.500 | 10026.000 | 10149.000 |
| Z | -2.319 | -2.839 | -.667 | -1.342 | -1.812 | -1.188 |
| Asymp. Sig. (2-tailed) | .020 | .005 | .505 | .180 | .070 | .235 |

1. Importance

|  | Name of Condition | Description of Condition | Purpose of Session | Goal of Session | Definition of Important Genetic Terminology | Family Dynamics |
| --- | --- | --- | --- | --- | --- | --- |
| Mann-Whitney U | 1043.500 | 827.000 | 1560.000 | 1330.000 | 1100.500 | 1174.500 |
| Wilcoxon W | 9428.500 | 9212.000 | 9945.000 | 9586.000 | 9485.500 | 9559.500 |
| Z | -5.960 | -6.792 | -4.068 | -4.879 | -5.755 | -5.493 |
| Asymp. Sig. (2-tailed) | .000 | .000 | .000 | .000 | .000 | .000 |

**Supplemental Data 7**. Mann-U Whitney Outputs Comparing Genetic Counselor and Healthcare Interpreter Perspectives on Roles within a Genetic Counseling Session

1. Advocacy

|  | Empowering Patients | Clarifying patient understanding | Improving patient health literacy | Advocating for patient |
| --- | --- | --- | --- | --- |
| Mann-Whitney U | 2299.500 | 2010.500 | 2405.000 | 1599.500 |
| Wilcoxon W | 10555.500 | 10138.500 | 10533.000 | 9855.500 |
| Z | -.782 | -2.179 | -.286 | -3.780 |
| Asymp. Sig. (2-tailed) | .434 | .029 | .775 | .000 |

1. Psychosocial

|  | Assessing patient affect | Managing patient emotions | Expressing empathy verbally | Expressing empathy nonverbally | Engaging in relationship-building with the patient |
| --- | --- | --- | --- | --- | --- |
| Mann-Whitney U | 1968.500 | 1913.000 | 2124.500 | 2463.500 | 2477.500 |
| Wilcoxon W | 10224.500 | 10169.000 | 10380.500 | 10719.500 | 3257.500 |
| Z | -2.116 | -2.402 | -1.708 | -.414 | -.075 |
| Asymp. Sig. (2-tailed) | .034 | .016 | .088 | .679 | .940 |

1. Cultural

|  | Ensuring the content is delivered in a culturally-appropriate way | Maintaining understanding of patient's culture | Reacting to patient cultural concerns | Assessing relevant patient cultural beliefs |
| --- | --- | --- | --- | --- |
| Mann-Whitney U | 1450.000 | 1258.500 | 1463.500 | 1168.000 |
| Wilcoxon W | 9706.000 | 9514.500 | 9719.500 | 9424.000 |
| Z | -4.506 | -5.643 | -4.481 | -5.470 |
| Asymp. Sig. (2-tailed) | .000 | .000 | .000 | .000 |
