# Supplementary material for: Exploring United States genetic counselor and healthcare interpreter perspectives: Allocation of roles within the genetic counseling encounter
Source: J Genet Couns. 2022 Apr 13;31(4):976–88. doi: 10.1002/jgc4.1572 (PMC9542924; doi:10.1002/jgc4.1572)
Supplement: Supplementary file 7 — Data S8 [file JGC4-31-976-s005.docx]

**Supplemental Data 8.** Genetic Counselor and Healthcare Interpreter Free-Text Response Themes and Representative Quotes on Suggestions to Improving the Working Relationship.

| **Theme** | **Genetic Counselor Quotes** | **Healthcare Interpreter Quotes** |
| --- | --- | --- |
| **Integrating Pre-session** | “Providing additional information to the interpreter as far as terminology you're going to be using and the definitions of such language upfront.” | “Staff interpreters are able to access the electronic health record to prepare for an assignment, but having a quick briefing on the session ahead of time would be helpful.” |
| **Collaboration between GC and HI** | “I think the genetic counselor and interpreter should collaborate, as the interpreter has potential insight into the culture/understanding of the patient based on tone/non-verbal and verbal cues that might not be fully communicated in the direct translation.” | “I would like to consider myself as interpreter at first but would also like to advocate for patients always. I would like the suggest Genetic counselor to get some cultural background and ideas from the interpreters as well it will be easy to understand and explore more about the patient” |
| **Increased education for both GCs and HIs on working with each other** | “Additional education on both sides (GC to HI and HI to GC). Certain language have no concept of genetics in their language, so knowing this upfront can be helpful in only using analogy statements or other useful concepts in their own language.”  “More practical training (such as tips on pre-session communication) provided in GC training programs”  “Increased awareness for GC's on ways in which HI want to be engaged in the session.” | “Required training for interpreters.  There is no standard generally applied to interpreter skill and knowledge base which makes it difficult for genetics counselors to understand what they should be able to expect from an interpreter.  More orientation for genetic counselors on the ethics and standards of practice for interpreters and the issues of message conversion.” |
| **Specific HIs for Genetics** | “Specific interpreters assigned to Genetics. That way they can get more familiar with some of the terminology and not as much background would be [necessary]. We call into a large interpreter service and I rarely get the same interpreter again.”  “Having continuity of interpreter and counselor between pre and post-test sessions.” | N/A |
| **Increased exposure to each other** | “The more you work with interpreters the most comfortable you become and the better you are as a partner in the care of your patient. More exposure is the way to make the relationship better and the patient experience better.” | N/A |
| **Written information in the patient’s language** | N/A | “The Genetic counselor should provide some of the material to family in the language they can read. (Translation of written material)  The Lab agreement form is too long and detail related to laws and other terminology. 'Significant' and the use of the term mutation etc...” |
| **GC utilizing plain language** | N/A | “Practice, learn, discuss how to explain terms to someone who has never been to school and has no idea what a cell is, let alone DNA, gene, chromosomes, sperm, egg, etc.”  “Genetic counselor should use plain language whenever possible and when not possible, explain the high register term in living-room language. Use pictures to show relationships and explain concepts.” |
